# Telomerase reverse transcriptase activates transcription of *miR500A* to inhibit Hedgehog signalling and promote cell invasiveness

**DOI:** 10.1002/1878-0261.12943

**Published:** 2021-05-02

**Authors:** Manuel Bernabé‐García, Elena Martínez‐Balsalobre, Diana García‐Moreno, Jesús García‐Castillo, Beatriz Revilla‐Nuin, Elena Blanco‐Alcaina, Victoriano Mulero, Francisca Alcaraz‐Pérez, María L. Cayuela

**Affiliations:** ^1^ Telomerase, Cancer and Aging Group Research Unit Department of Surgery University Hospital ‘Virgen de la Arrixaca’ Murcia Spain; ^2^ Instituto Murciano de Investigación Biosanitaria (IMIB‐Arrixaca) Murcia Spain; ^3^ CIBERER, Instituto de Salud Carlos III Madrid Spain; ^4^ Department of Cell Biology and Histology Faculty of Biology University of Murcia Spain

**Keywords:** cancer, Hedgehog, mircoRNAs, telomerase, therapeutic strategies, zebrafish xenografts

## Abstract

Telomerase reverse transcriptase (TERT) maintains telomere homeostasis, thus ensuring chromosome stability and cell proliferation. In addition, several telomere‐independent functions of human TERT have been described. In this study, we report that TERT binds directly to the TCF binding elements located upstream of the oncomiR miR500A, and induces its transcription. This function was independent of the telomerase activity, as shown with experiments using catalytically inactive TERT and inhibitors of TERT and the TERT RNA component. miR500A was in turn found to target three key components of the Hedgehog signalling pathway: Patched 1; Gli family zinc finger 3; and Cullin 3, thereby promoting cell invasion. Our results point to the crucial role of the TERT–miR500A–Hedgehog axis in tumour aggressiveness and highlight the therapeutic potential of targeting noncanonical TERT functions in cancer.

## Graphical abstract

Human telomerase (TERT) is reactivated in approximately 90% of all cancers. Upon reactivation, TERT binds to the promoter region and induces the transcription of *miR500A* in tumour cells. *miR500A* induces the Hedgehog signalling pathway by regulating the mRNA degradation of *PTCH1*, subsequently promoting cancer cell invasion. Our study describes a novel, noncanonical function of TERT and highlights the therapeutic potential of targeting noncanonical TERT functions in cancer.

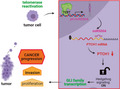

**Abbreviations**
ALT: alternative lengthening of the telomere
BLCA: bladder urothelial carcinoma
CLCN5: Chloride voltage‐gated channel 5
CUL3: Cullin 3
DN‐TERT: dominant‐negative mutant of TERT
GLI1/2/3: Gli family zinc finger
HEK293: human embryonic kidney 293
HeLa cells: Henrietta Lacks cells (cervical cancer)
Hh: Hedgehog signalling pathway
PNA: peptide nucleic acid
PTCH: Patched 1
SAOS 2: osteosarcoma cell line
STAC: stomach adenocarcinoma
TBE: TCF binding elements
TCGA: The Cancer Genome Atlas

## 1 Introduction

Human telomerase (TERT) is reactivated in approximately 90% of all cancers, while in approximately 10% of tumours, telomere length is maintained, independently of TERT, by the homologous recombination‐mediated alternative lengthening of the telomere (ALT) pathway [1]. Increasing evidence points to the noncanonical roles of TERT, not only in cancer but also in several essential cellular functions, *via* mechanisms that are independent of telomere maintenance. These novel roles of TERT may endow transformed cells with specific capacities in many stages of tumour development [2]. Among its numerous nontelomeric biological functions, TERT has been demonstrated to act as a regulatory molecule, modulating gene transcription [3, 4, 5, 6] and cell proliferation [7]. However, the noncanonical roles of TERT in cancer and their relevance in its progression and response to therapy remain poorly understood.

miRNAs are endogenous noncoding small RNAs (~ 22 nucleotides) that cause post‐transcriptional repression or cleavage of target mRNAs by binding to their 3′UTR. Around 50% of all miRNA genes are located within 50 kb of another miRNA gene and are transcribed together as a cluster, frequently with a similar sequence homology in the seed sequence (the region for target recognition), providing identical targets for a miRNA cluster [8]. Several studies have estimated that each miRNA can regulate more than 200 genes [9, 10], which suggests that miRNAs regulate a large number of biological processes that are frequently altered in many human diseases. Over the past 15 years, accumulated evidence has shown not only that aberrant miRNA expression is involved in tumorigenesis and metastasis [11] but also that the miRNA expression profile is unique to each cancer type, suggesting that blood‐based miRNA expression patterns can be used as a noninvasive method for cancer diagnosis [12]. In 2014, Drevytska and colleagues showed a positive correlation between the expression of *TERT* and several miRNAs [13]. Consistent with these results, gastric cancer models revealed that TERT regulates several miRNAs [14]. However, the regulatory mechanisms involved are not fully understood.

Despite the significant clinical advances that have been made, the mortality associated with most solid tumours throughout the world is largely due to metastasis, a highly dynamic process that occurs in multiple steps regulated by several signalling pathways. However, metastasis is still an incompletely understood process, especially the initial steps that lead to intravasation, when small developing tumours and micrometastases are not easily detected [15]. Thus, there is a crucial need to understand the invasive mechanisms involved and angiogenic programmes that facilitate metastasis so that therapeutic strategies can be developed to block disease progression.

Because TERT has a noncanonical role in regulating the expression of genes involved in cancer initiation and dissemination, the present study aimed to identify the miRNAs regulated by TERT and then use a zebrafish xenograft model [16] to investigate the above‐mentioned noncanonical role of TERT in metastasis by regulating these miRNAs. It was found that TERT directly regulates *miR500A* by binding to the TCF binding elements (TBEs) located upstream of this gene, which results in inhibition of the Hedgehog signalling pathway and increased tumour invasiveness. The results throw light on the noncanonical role of TERT in promoting cancer invasiveness and reveal novel targets for therapeutic intervention.

## 2 Methods

### 2.1 Animals

Zebrafish (*Danio rerio* H., Cypriniformes, Cyprinidae) were obtained from the Zebrafish International Resource Center and mated, housed, raised and processed using standard procedures. Details of husbandry and environmental conditions are available at protocols.io (https://doi.org/10.17504/protocols.io.mrjc54n).

The performed experiments comply with the Guidelines of the European Union Council (86/609/EU). Experiments and procedures were performed as approved by the Bioethical Committee of the University Hospital ‘Virgen de la Arrixaca’ (HCUVA Spain).

### 2.2 Cell culture

Human embryonic kidney 293 (HEK293), cervical cancer (HeLa 1211) and sarcoma osteogenic (SAOS 2) cell lines were purchased from the ATCC (#CRL‐1573.3, #CCL‐2 and #HTB‐85, respectively). All cell lines were maintained in Dulbecco's Modified Eagle Medium (Sigma, Madrid, Spain, #D‐5796) supplemented with 10% FBS (Biowest, Nuaillé, France, #S1810‐500), and were cultured at 37 °C with 5% CO_2_.

pBABE‐SAOS 2 and hTERT‐SAOS 2 stable cell lines were obtained after transfection of the SAOS 2 cell line with the plasmids pBABE‐puro or pBABE‐puro‐hTERT from Addgene (Teddington, UK; #1764 and #1771, respectively) and Lipofectamine 2000 (Invitrogen, Madrid, Spain, #11668‐027) following the manufacturer's instructions. Then, several stable clones were selected with puromycin.

### 2.3 Cell proliferation assay

Cell proliferation assays were performed using the Cell Proliferation Reagent WST‐1 (Roche, Basel, Switzerland) following the manufacturer's instructions. Briefly, cells were seeded into 96‐well plates. At the indicated times, WST‐1 reagent was added to the well and cells were further incubated at 37 °C for 4 h. Absorbance at 450 and 620 nm was measured, and the proliferation rate at each time point was determined as follows: (Abs450‐Abs620)/(Abs450‐Abs620)T0.

### 2.4 Western blot

Cells were harvested, washed twice in PBS and lysed in RIPA buffer (50 mm Tris/HCl, pH 7.4, 150 mm NaCl, 1 mm EDTA, 0.5 mm DTT, 0.1% SDS, 1% NP‐40, 0.5% sodium deoxycholate) containing a protease inhibitor cocktail (Sigma). The samples were then incubated for 30 min on ice with frequent vortexing and centrifuged for 20 min at 4 °C, followed by determining the protein concentration of the supernatant. The proteins were subjected to polyacrylamide gel electrophoresis, transferred to nitrocellulose membranes (Bio‐Rad, Madrid, Spain) and blotted using 1 : 1000 anti‐TERT rabbit antibody (Rockland, Limerick, PA, USA) and 1 : 5000 anti‐μ‐actin HRP‐conjugated mouse monoclonal antibody (C4; Santa Cruz Biotechnology, Heidelberg, Germany).

### 2.5 Zebrafish xenograft assay

Cells were trypsinized, washed and stained with the vital cell tracker red fluorescent CM‐Dil (4 ng·μL^−1^ final concentration; Invitrogen, #C7001), following the manufacturer's instructions. Zebrafish larvae, previously treated with PTU (*N*‐phenylthiourea; Sigma‐Aldrich, #222909) to inhibit the skin pigmentation, were dechorionated and anaesthetized with tricaine (Sigma, #A5040). Then, 100–150 labelled cells in 4 nL were injected into the yolk sac of 2 dpf zebrafish larvae using a manual injector [Narishige IM‐300, East Meadow (Long Island), NY, USA]. After injection, embryos were incubated for 2 h at 31 °C and checked for cell presence 2 hours postxenograft (hpx). Fish with fluorescent cells outside the implantation area at 2 hpx were excluded for further analysis. All other fish were incubated at 35 °C for 48 h and analysed with a SteReo Lumar.V12 stereomicroscope fitted with an AxioCam MR5 camera (Carl Zeiss, Thornwood, NY, USA). The evaluation criterion for invasion was at least three cells outside the yolk.

### 2.6 miRNA microarray

RNA from 18 nucleotides (nt) upwards was isolated from two different clones of pBABE‐SAOS 2 and hTERT‐SAOS 2 stable cell lines by miRNeasy Mini Kit (Qiagen, Hilden, Germany, #79306), following the manufacturer's instructions. A total of 0.5 µg of RNA from each sample were sent to the CNIO Genomic Facility. For quality control, all samples were analysed on a NanoDrop instrument (Bioanalyzer 2100; Agilent, Santa Clara, CA, USA), labelled and hybridized on the Human miRNA 8x15K, 1 colour array (Agilent, #G4470C). These arrays contained probes for 2689 microRNAs. Data analysis was performed, and a gene list according to a *P*‐value < 0.05 was obtained.

### 2.7 Gene and microRNA expression analysis

RNA from 18 nt upwards was extracted from 106 cells homogenized in QIAzol Lysis Reagent (Qiagen, #79306) using the miRNeasy Mini Kit (Qiagen, # 217004), following the manufacturer's instructions. cDNA was generated by the miScript II RT Kit (Qiagen, #218161), following the manufacturer's instructions, and treated with DNase I, amplification grade (1 U·µg^−1^ RNA, Qiagen, #79254). Real‐time qPCR was performed with a MyiQ™ instrument (Bio‐Rad), using the miScript SYBR Green PCR Kit (Perfect Real Time; Qiagen, #218161). Reaction mixtures were incubated for 10 min at 95 °C, followed by 40 cycles of 15 s at 95 °C, 1 min at 60 °C, and finally 15 s at 95 °C, 1 min at 60 °C and 15 s at 95 °C. For each sample, microRNA or gene expression was normalized to the U6 snRNA or GAPDH content in each sample, respectively, using the comparative Ct method (2^−ΔΔCt^). The primers used are shown in Table S1. In all cases, each PCR was performed with triplicate samples and repeated with two independent samples at least.

### 2.8 Overexpression experiments

TERT (Addgene plasmid #1771) and a dominant‐negative mutant (DN‐TERT) (Addgene plasmid #1775), and different members of the MIR500 cluster (pre‐MIR532, pre‐MIR500A, pre‐MIR362 and pre‐MIR502, from Ambion, Madrid, Spain, #PM11553, #PM12793, #PM10870 and #PM10480, respectively) were overexpressed in pBABE‐SAOS 2 and hTERT‐SAOS 2 after transfection with Lipofectamine 2000 following the manufacturer's instructions. Forty‐eight hours after transfection, cells were trypsinized and split to carry out functional assays and to measure the expression level by real‐time RT‐PCR analysis.

### 2.9 Silencing experiments

To inhibit the *miR500A*, a specific peptide nucleic acid (PNA) miRNA inhibitor was used (Panagene, Daejeon, South Korea, #PI‐1487‐FAM). The PNA miRNA inhibitor was preheated for 10 min at 70 °C, and cells were then transfected with a final concentration of 500–2000 nm by using Lipofectamine 2000, following the manufacturer's instructions.

To silence the telomerase, HEK293 cells were transfected with a ready‐to‐use siRNA for human TERT [TERT siRNA (h), Santa Cruz Biotechnology, #sc‐36641] or for human Patched 1 (PTCH1; PTCH1 siRNA (h), Santa Cruz Biotechnology, #sc‐91304), at a final concentration of 20 mm using Lipofectamine 2000, according to the manufacturer's instructions. Forty‐eight hours after transfection, cells were trypsinized and split for functional assays and to measure the knockdown efficiency by real‐time RT‐PCR analysis.

### 2.10 Analysis of *miR500A* promoter activity

A 2 Kb genomic DNA sequence upstream of *miR500A* + 1 position was amplified using the primers: forward 5′CAGTGTTGTGGTTTTGGTCCAGGCG3′ and reverse 5′CCGGACACCGAGCACCGGCGAGCCGCC3′. The DNA fragment was cloned in the SmaI site of the pGL3‐Basic Vector (Promega, Madison, WI, USA, #E1761) to drive the expression of firefly luciferase reporter gene (p*miR500A*‐Luc). Cells were transfected with a mixture containing 100 ng of the firefly luciferase construct and 50 ng·µg^−1^ of Renilla luciferase control plasmid, using Lipofectamine 2000, according to the manufacturer's instructions. After 48 h, cell extracts were assayed for luciferase activity by using the Dual‐Luciferase Assay Kit (Promega, #E1910), as specified by the manufacturer, in an Optocomp I luminometer (MGM Instruments, Hamden, CT, USA).

### 2.11 ChIP assay

Both pBabe‐SAOS 2 and hTERT‐SAOS 2 (107 cells) were cross‐linked with 1% paraformaldehyde (Sigma‐Aldrich, # P6148) in culture medium for 10 min at room temperature. Then, the aldehydes were quenched with PBS containing 200 mm glycine (Sigma‐Aldrich, #M6635) for 5 min followed by a PBS wash. The cells were centrifuged at 200 ***g*** for 10 min at 4 °C to pellet. Then, they were resuspended in lysis buffer containing protease inhibitors (Sigma‐Aldrich, #P2714) and the lysate was sonicated using a Bioruptor Plus Sonication System (Diagenode, Ougree, Belgium) for 30 cycles of 30 s ON, 30 s OFF. The sonicated lysate was centrifuged at 20 000 ***g*** for 10 min at 4 °C, and the supernatant was transferred to new tubes. ChIP dilution buffer (100 µL) was added to the supernatant, and 10 μL of this supernatant was set aside for input. The chromatin was bound to the Antibody–Dynabeads complexes, the formaldehyde crosslinking of the chromatin was reversed, and the DNA was purified, as described in the protocol MAGnif ChIP System (Invitrogen, #49024).

The ChIP results were analysed by real‐time qPCR, with a qPCR ABI PRISM 7500 instrument (Applied Biosystems, Madrid, Spain), using the commercial kit Power SYBR Green PCR Master Mix (Applied Biosystems, #4309155). Reaction mixtures were incubated for 15 min at 95 °C, followed by 40 cycles of 15 s at 94 °C, 30 s at 55 °C, and finally 30 s at 70 °C, 1 min at 95 °C and 1 min at 60 °C. The primers used are shown in Table S2.

### 2.12 Quantitative telomerase activity assay, Q‐TRAP

To quantitatively measure the telomerase activity, total proteins were extracted from cells using ice‐cold CHAPS lysis buffer (Sigma‐Aldrich, #S7705) and real‐time Q‐TRAP performed with 0.1 µg protein extracts. A negative control of each sample confirmed the specificity of the assay (data not shown in figure). Control samples were obtained by treating the cell extracts with 1 µg RNase (Thermo Fisher, Madrid, Spain, #EN0531F) at 37 °C for 20 min. To make the standard curve, a 1 : 10 dilution series of telomerase‐positive sample (HeLa cells) was used. After qPCR amplification, real‐time data were collected and converted into relative telomerase activity (RTA) units by means of the following calculation: RTA of sample  =  10(Ct sample − Yint)/slope. The standard curve obtained was given by: *y*  = 23.802  − 3.2295*x*.

### 2.13 Chemical inhibition of telomerase activity

BIBR 1532 (Santa Cruz Biotechnology, #sc‐203843) and TAG‐6 (Calbiochem, Merck, Madrid, Spain, #581004) were added to make a final concentration of 20 and 2.5 µm in cell cultures, respectively. As a control, DMSO was adjusted to the same concentration. Cells were incubated with the compounds for 15 h, then trypsinized and divided for xenografting, checking telomerase activity by Q‐TRAP and studying gene expression by real‐time RT‐PCR.

### 2.14 *miR500A* target prediction and validation

We have used the TargetScan database (http://www.targetscan.org) to predict the potential targets. Then, we validated the chosen targets by real‐time RT‐qPCR and luciferase experiments. To validate the specific binding of *miR500A* to the PTCH1 3′UTR, a 1.2‐Kb genomic DNA 3′UTR sequence of PTCH1 was amplified using the primers: forward 5′AAGGTCTAGAGCAAAGAGGCCAAAGATTGGA3′ and reverse 5′TCTAGAAAGCCTCAACCAGC3′. We also amplified the same region but lacking the *miR500A* binding site using the primers: forward 5′AATATTGCTTATGTAA TATTATTTTGTAAAGG3′ and reverse 5′CCTTTACAAAATAATATTACATAAG CAATATT3′. The 3′UTR fragments were cloned in the XbaI site of the pRL‐CMV Vector (Promega, #E2261; pCMV‐Luc‐PTCH1 3′UTR wt/mut). Cells were transfected, and luciferase experiments were performed as explained above.

### 2.15 Statistical analysis

All data are expressed as the mean ± standard error of mean (SEM). Values of *P* < 0.05 were considered statistically significant. Statistical analyses were performed using analysis of variance (ANOVA) followed by different post hoc comparison tests. The differences between two samples were analysed by Student's *t*‐test. The percentage of zebrafish larvae that had been invaded was analysed by the chi‐square test (Fisher's exact test). The correlation among the gene expression data from the The Cancer Genome Atlas (TCGA) Research Network was analysed using Spearman's correlation. All analyses were performed with graphpad prism 7 (GraphPad company, San Diego, CA, USA).

## 3 Results

### 3.1 TERT expression increases tumour cell line invasion

**Fig. 1:**
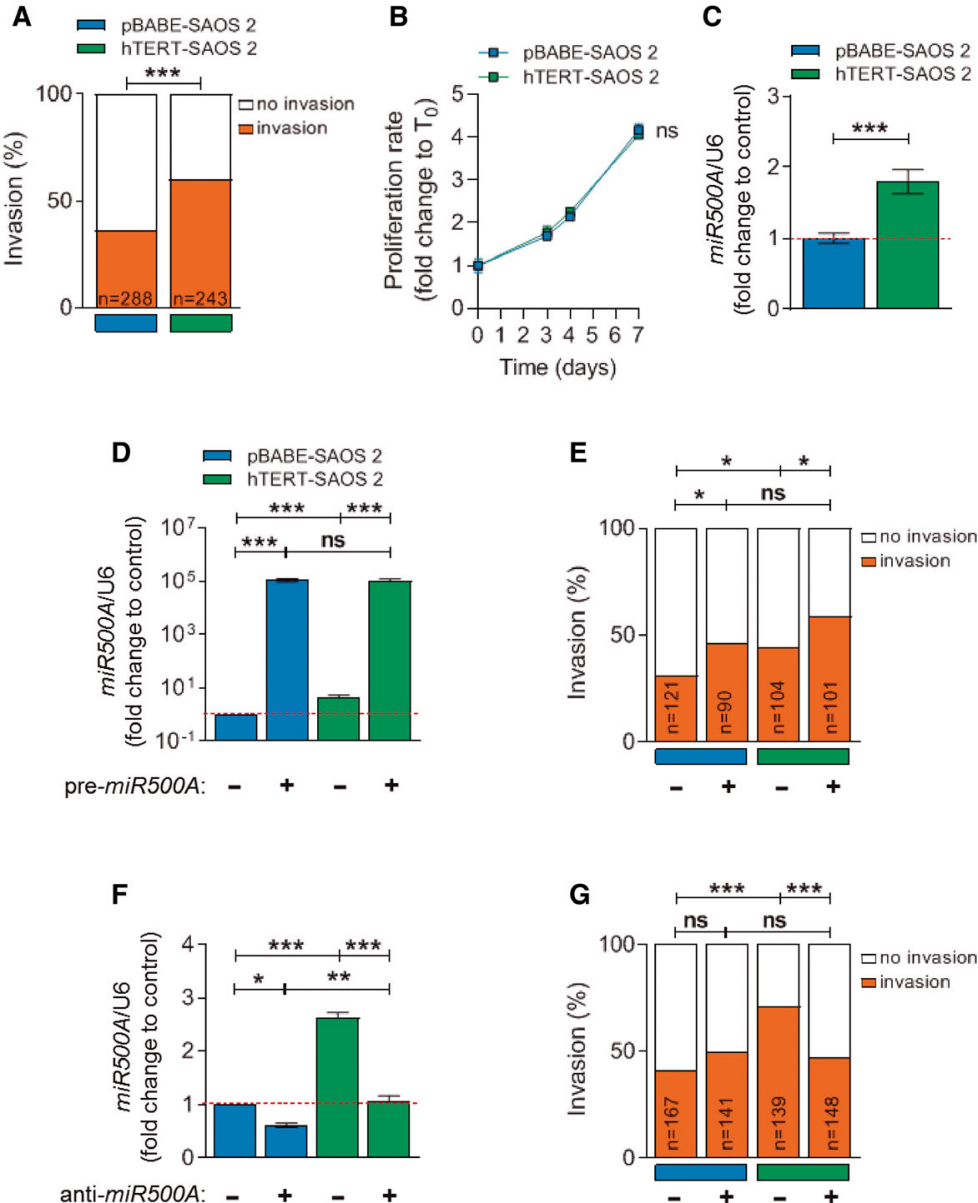
TERT up‐regulates the expression of *miR*500A, leading to an increase in the *in vivo* invasive capacity. (A) Quantification of the *in vivo* invasive capacity and (B) proliferation rate of SAOS 2 cells. (C) Quantification of *miR500A* levels in TERT overexpression conditions by real‐time RT‐qPCR. (D‐G) Overexpression and inhibition of *miR500A* by transient transfection with the *pre‐miR500A* (D, E) or with an *anti‐miR500A* PNA probe (F, G), respectively, in both pBABE‐ and hTERT‐SAOS 2 cells. (D, F) Quantification of *miR500A* level and (E, G) its effect on the *in vivo* invasive capacity. In (C, D, F), each bar represents the mean ± SEM from triplicate samples. In (A, E, G), histogram represents the accumulative value of the invasion percentage of the number of larvae stated in the figure for each treatment. Graphs are representative of three (*N* = 3) (C, D, F). ns, not significant; *P < 0.05; **P < 0.01; ***P < 0.001 according to Student's *t*‐test (C), ANOVA followed by Tukey's multiple comparison test (D, F) and Fisher's exact test (A, E, G).

To study the noncanonical functions of telomerase in invasiveness, the cell line SAOS 2, a telomerase‐negative osteosarcoma cell line that maintains its telomeres *via* ALT, was stably transfected with the plasmid pBABE‐puro‐hTERT. Two clones with high *TERT* expression (Fig. S1A) were selected for zebrafish larva xenotransplantation assays (Fig. S1C,D). Sixty per cent of the zebrafish larvae injected with the hTERT‐SAOS 2 line had cells outside the yolk sac, whereas only 40% of the larvae had invasion after injection of the parental cells pBABE‐SAOS 2 (Fig. 1A, Fig. S1B). The proliferation of pBABE‐SAOS2 and hTERT‐SAOS2 cell lines was measured, since emerging noncanonical roles of TERT include the regulation of cell cycle. However, no differences in the proliferation index of the parental and TERT‐overexpressing cell lines were observed (Fig. 1B). Therefore, exogenous *TERT* expression in SAOS 2 increased their invasiveness *in vivo* but not its proliferation.

### 3.2 TERT regulates the expression of miR500A

To evaluate whether the TERT‐dependent invasiveness of cancer cells is mediated through the regulation of miRNAs, a miRNA microarray was used to analyse the miRNA expression profile in TERT overexpression conditions, finding that only the oncomiR *miR500A* was significantly up‐regulated. We verified this result by RT‐qPCR (Fig. 1C). To confirm that the higher invasiveness of TERT‐expressing tumour cells was mediated by *miR500A*, its expression levels in pBABE‐SAOS 2 cells were manipulated by transfecting with pre*‐miR500A* or with a PNA‐labelled anti‐*miR500A* probe. Strikingly, *miR500A* overexpression increased the *in vivo* invasive capacity of both control and TERT‐expressing SAOS 2 cells (Fig. 1D,E), while *miR500A* inhibition specifically reduced the increased invasiveness of hTERT‐SAOS 2 cells (Fig. 1F,G). Similarly, genetic inhibition of TERT in telomerase‐positive HeLa cells resulted in reduced expression levels of *miR500A* and impaired invasiveness (Fig. S2A–C).

### 3.3 TERT regulates the MIR500 cluster by directly binding to its promoter region

**Fig. 2:**
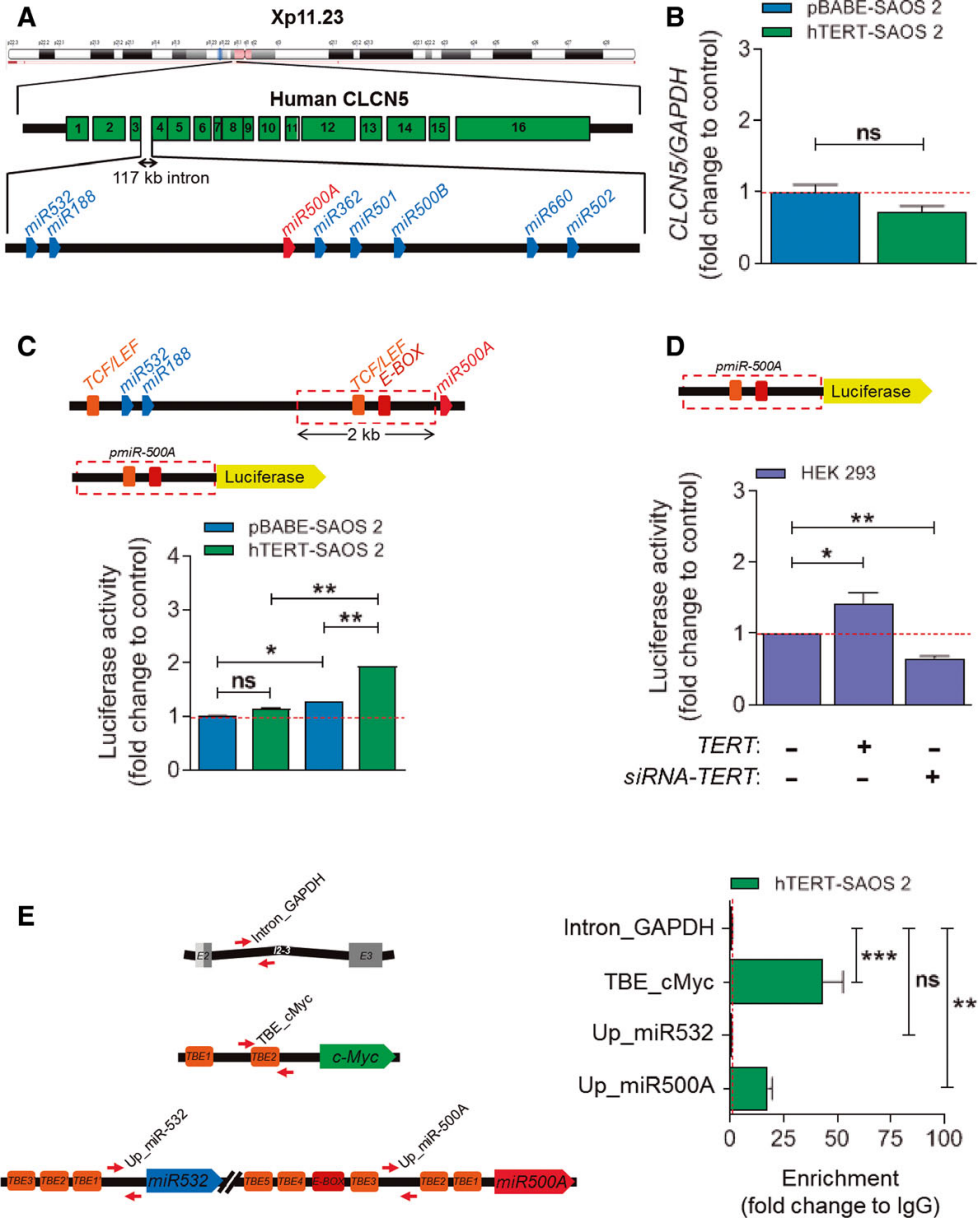
TERT regulates *miR500A* by directly binding to its promoter region. (A) Schematic representation of the *miR*500 cluster according to the Ensembl database. Names are shortened to simplify. (B) Quantification of *CLCN5* mRNA levels in TERT overexpression conditions by real‐time RT‐qPCR. (C) *miR500A* promoter activity in TERT overexpression conditions and (D) in TERT‐depleted HEK293 cells by luciferase reporter assay. (E) Determination of the promoter occupancy by amplification using a ChIP assay in hTERT‐SAOS 2 cells. The schemes represent the primers mapping to both negative (intron_GAPDH) and positive (TBE_cMyc) controls, and to the *miR*500 cluster. Each bar represents the mean ± SEM from triplicate samples. Graphs are representative (B, E) or the average (C, D) of three (*N* = 3) (B‐D) or two (*N* = 2) (E) independent experiments. ns, not significant; *P < 0.05; **P < 0.01; ***P < 0.001 according to Student's *t*‐test (B, C) and ANOVA followed by Dunnett's multiple comparison test (D, E).

According to the *Ensembl* database (https://www.ensembl.org), the oncomiR *miR500A* is located in a cluster of eight miRNAs, which is called the MIR500 cluster, in the short arm of the human X chromosome (Xp11.23) and within the intron 3 of the Chloride voltage‐gated channel 5 (*CLCN5*) gene (Fig. 2A). Although the majority of intronic miRNAs are transcribed from the same promoter as the host gene, approximately one‐third of them are transcribed from independent promoters, enabling separate control of their transcription [17]. For this reason, we studied whether or not the expression of the *CLCN5* gene was affected by TERT, finding that that the expression was similar in parental and TERT‐expressing SAOS 2 cells (Fig. 2B). To address the mechanism by which TERT regulates the transcription of *miR500A*, we cloned the 2‐Kb upstream sequence of *MIR500A* into a luciferase reporter plasmid. According to the PROmiRNA database, (https://tools4mirs.org/software/other_tools/promirna/), this sequence contains several TBEs. The luciferase reporter assay showed that TERT was able to increase the expression of the reporter driven by the 2‐Kb upstream sequence of *miR500A* (Fig. 2C). These results were further confirmed in HEK293 cells, a telomerase‐positive cell line, in which the overexpression of hTERT increased and inhibition by siRNA decreased the *miR500A* promoter activity (Fig. 2D, Fig. S3).

The luciferase reporter results prompted us to investigate, by means of ChIP experiments, whether TERT occupied the *miR500A* promoter. TERT was seen to be associated with the region upstream of *miR500A* but failed to bind the upstream sequence of *miR532*, which also contains several TBE (Fig. 2E). As expected, TERT was also seen to bind to the TBE found upstream of the oncogene *MYC*, as previously shown [3].

**Fig. 3:**
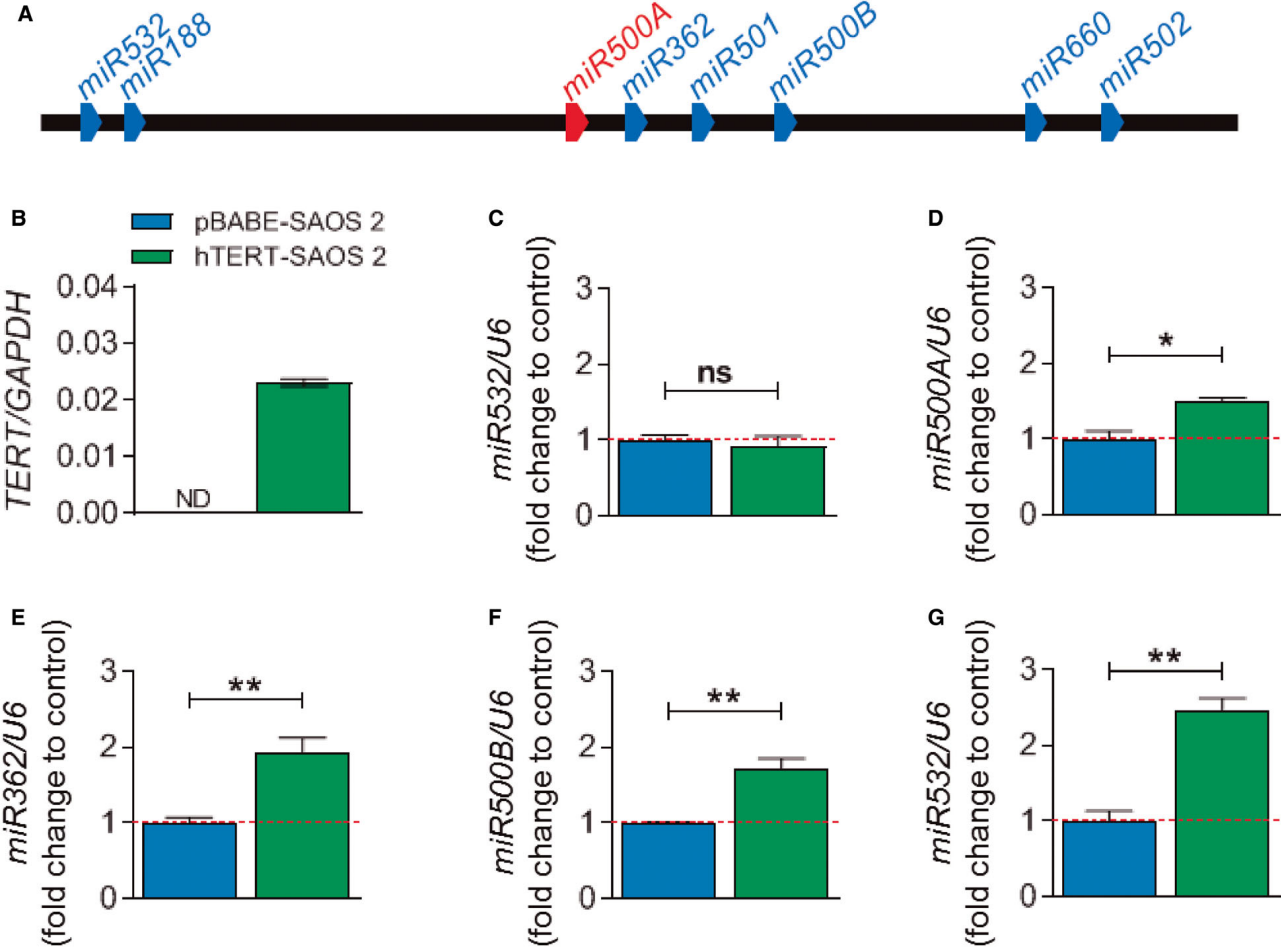
The *miR*500 cluster is regulated by TERT. (A) Schematic representation of the *miR*500 cluster. (C–G) Quantification of the mRNA levels of five different *miR*NAs from the *miR*500 cluster under TERT overexpression conditions (B) by real‐time RT‐qPCR. Each bar represents the mean ± SEM from triplicate samples, and graphs are representative of three different experiments (*N* = 3). ND, not determined; ns, not significant; *P < 0.05; **P < 0.01 according to Student's *t*‐test.

These results suggest that the whole *miR*500 cluster may be regulated by TERT. The RT‐qPCR analysis of parental and TERT‐expressing SAOS 2 (Fig. 3A) revealed that TERT induced the expression of *miR500A*, *miR362, miR500B* and *miR502* (Fig. 3D–G) but did not affect the expression of *miR532* (Fig. 3C) and was unable to bind its upstream TBE.

### 3.4 miR500A mediates TERT‐driven invasiveness of tumour cells

**Fig. 4:**
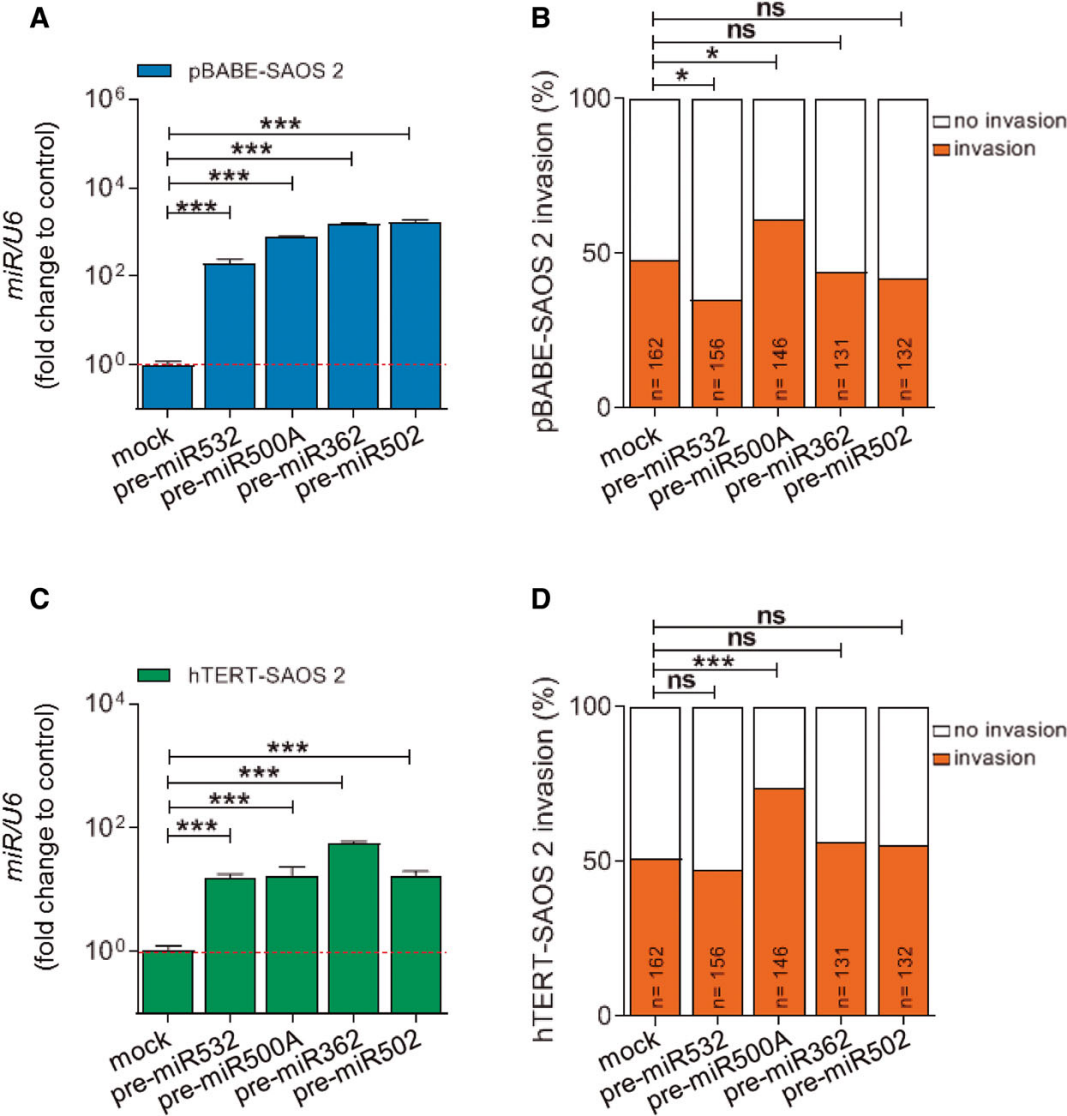
Only the *miR500A* is able to increase the invasiveness. Contribution of the different miRNAs from the *miR*500 cluster to the *in vivo* invasion capacity of pBABE‐SAOS 2 (A, B) and hTERT‐SAOS 2 (C, D) cells. In (A, C), each bar represents the mean ± SEM from triplicate samples. In (B, D), histograms represent the accumulative value of invasion percentage of the number of larvae stated in the figure for each treatment. Graphs are representative (A, C). ns, not significant; *P < 0.05; ***P < 0.001 according to ANOVA followed by Dunnett's multiple comparison test (A, C) and Fisher's exact test (B, D).

We next examined whether the different components of the cluster are also involved in TERT‐mediated tumour invasion. For this, several *miR*NAs were overexpressed in parental and TERT‐expressing SAOS 2 cells by transient transfection with the corresponding *pre‐miR* (Fig. S4) and the effect on the *in vivo* invasive capacity of these cells was studied (Fig. 4). Surprisingly, *miR500A* was the only one able to increase the *in vivo* invasion capacity of tumour cells, while the *miR532*, which is not regulated by TERT, inhibited the invasiveness of parental tumour cells (Fig. 4B,D). Taken together, these results show that *miR500A* mediates *TERT*‐driven tumour invasiveness.

### 3.5 The regulation of miR500A by TERT does not depend on telomerase activity

**Fig. 5:**
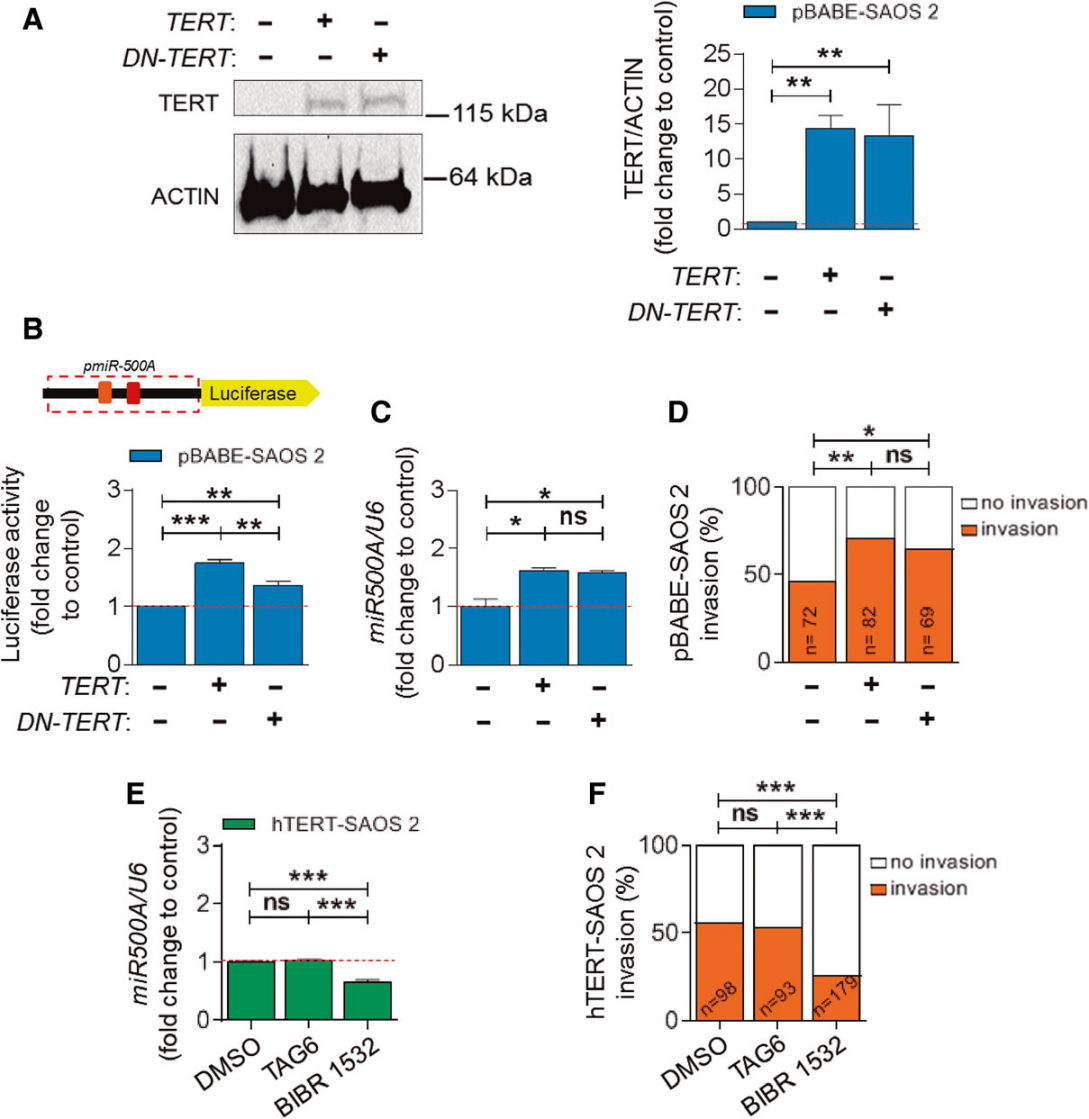
Telomerase activity is not involved in *miR500A* up‐regulation by TERT. (A–D) Transfection of pBABE‐SAOS 2 cells with *TERT* or *DN‐TERT* (protein levels showed and quantified in (A)) to determine whether telomerase activity is necessary for *miR500A* promoter activity (B), for TERT‐dependent *miR500A* expression (C) and for the *in vivo* invasive capacity (D). (E, F) Inhibition by specific chemicals of *TERC* and TERT subunits in hTERT‐SAOS 2 cell line and its effect on the levels of *miR500A* (E) and on the *in vivo* invasive capacity (F). Each bar represents the mean ± SEM from triplicate samples, and graphs are representative of two (*N* = 2) (A) or three (*N* = 3) (B, C, E) independent experiments. Histograms represent the accumulated value of invasion percentage of a total of larvae stated in the figure for each treatment. ND, not determined; ns, not significant; *P < 0.05; **P < 0.01; ***P < 0.001 according to ANOVA followed by Dunnett's (A), Tukey's (B, C, E), multiple comparison test and Fisher's exact test (D, F)

To ascertain whether TERT depends on its telomerase activity to regulate the expression of *miR*500A, a DN‐TERT was used, since it has two‐point mutations in the A motif of the RT domain that cause it to lack the enzymatic activity [18]. DN‐TERT was overexpressed at similar levels as wild‐type TERT (Fig. 5A) and also increased *miR*500A promoter activity (Fig. 5B), *miR*500A transcript levels (Fig. 5C) and tumour invasiveness *in vivo* (Fig. 5D). To further confirm the novel noncanonical role of TERT, we used two different chemical telomerase inhibitors: BIBR 1532, which binds and blocks the catalytic subunit, TERT [19]; and TAG 6, which binds and blocks the RNA component, TERC [20]. Although both chemicals were able to inhibit telomerase activity (Fig. S5A), only BIBR 1532 decreased both the expression of *miR*500A (Fig. 5E) and tumour invasiveness *in vivo* (Fig. 5F), indicating that the effect of BIBR 1532 was not due to its action on telomerase activity but due to its ability to block TERT transcriptional activity. As expected, and to ruling out any off‐target effect, the treatment of parental SAOS 2 cells with these drugs did not affect the expression of *miR500A* (Fig. S5B) or tumour invasiveness *in vivo* (Fig. S5C).

### 3.6 Hedgehog signalling pathway is regulated by miR500A

**Fig. 6:**
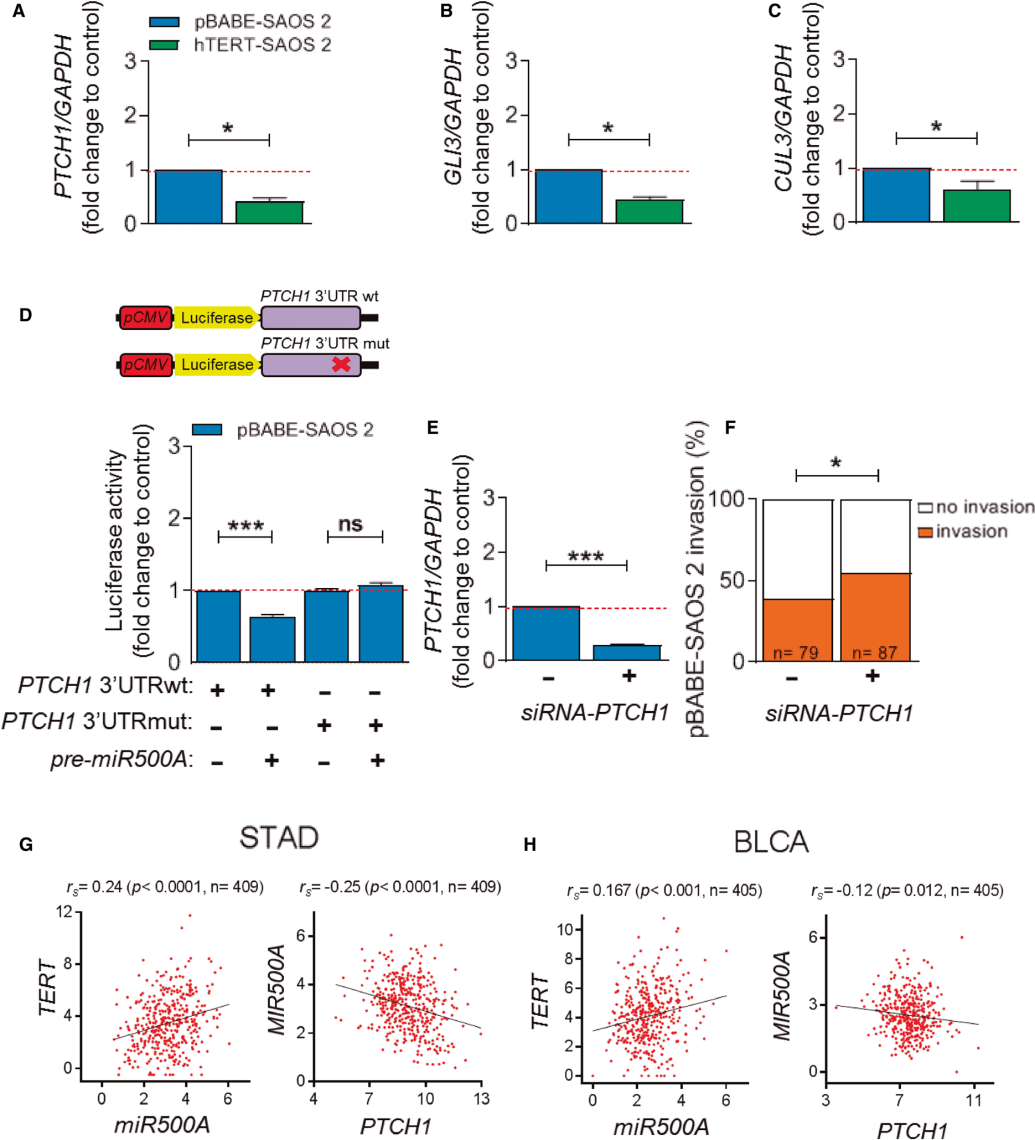
The Hedgehog signalling pathway is regulated by *miR500A*. (A‐C) Quantification of the mRNA levels of *PTCH1* (A), GLI3 (B) and CUL3 (C) by real‐time RT‐qPCR in TERT overexpression conditions. (D) Luciferase activity following cotransfection with *pre‐miR500A*, in combination with the indicated *PTCH1* 3′UTR constructs in cells. (E, F) Specific inhibition of *PTCH1* by *siRNA* in pBABE‐SAOS 2 cells. (E) Quantification of *PTCH1* level and (F) its effect on the *in vivo* invasive capacity. (G, H). Correlations between *TERT* vs *miR500A*, and *miR500A* vs *PTCH1* expression are shown in invasive STAC (G) and BLCA (H). Spearman's correlation (G, H) reveals a statistically significant positive correlation between *TERT* and *miR500A*, and a statistically significant negative correlation between *miR500A* and *PTCH1*. Each bar represents the mean ± SEM from triplicate samples, and graphs are the mean of three (*N* = 3) independent experiments (A–E). Histogram represents the accumulative value of the invasion percentage of the number of larvae stated in the figure for each treatment (F). ns, not significant; *P < 0.05; ***P < 0.001 according to Student's *t‐*test (A–C, E) and ANOVA followed by Bonferroni's multiple comparison test (D) and Fisher's exact test (F).

The *TargetScan* software (https://www.targetscan.org) revealed that the 3′UTR of 3253 human genes contains putative target sites for *miR500A*. Using the *MetaCore* software (https://www.omictools.com/metacore‐tool) to classify signalling pathways enriched in the predicted targets of *miR500A*, we identified the crucial role of several signalling pathways in cancer aggressiveness, such as Notch, Wingless and Hedgehog (Hh; Fig. S6A). Focusing on Hh pathway, since *PTCH1*, *GLI3* and Cullin 3 (*CUL3*) all have a putative target site for *miR500A* (Fig. S6B), real‐time RT‐qPCR confirmed that all three genes were down‐regulated in SAOS2 cells when TERT was overexpressed (Fig. 6A–6C, Fig. S7A). Notably, *GLI1* and *GLI2*, both members of the GLI family of zinc finger transcription factors (TFs), which lack putative target sites for *miR*500A, transcript levels were induced (Fig. S7B,C). In contrast, inhibition of TERT in HeLa cells, a telomerase‐positive cell line, led to increased transcript levels of *PTCH1* and *CUL3* genes (Fig. S2A–D,F), while those of Gli family zinc finger (GLI1/2/3) genes were hardly affected (Fig. S2E,G,H).

In addition, *miR500A* directly bound to *PTCH1* 3′UTR, as revealed by luciferase reporter experiments (Fig. 6D, Fig. S7D). Interestingly, PTCH1 inhibition by siRNA increased the invasiveness of SAOS2 cells, assayed by zebrafish xenotransplantation (Fig. 6E,F). Collectively, these results demonstrate the relevance of TERT‐*miR*500‐Hh axis.

The above results prompted us to look for any correlation among *TERT*, *miR500A* and *PTCH1* expression in different cancer histotypes. Analysis of data from TCGA Research Network (https://www.cancer.gov/tcga) revealed a significant positive correlation between the expression of *TERT* and *miR500A,* and a negative correlation between *PTCH1* and *miR500A* only in stomach adenocarcinoma (STAC) and bladder urothelial carcinoma (BLCA) (Fig. 6G,H). These results add weight to the relevance of inhibiting the extracurricular functions of telomerase in these specific cancer histotypes.

## 4 Discussion

Identifying and understanding the noncanonical functions of TERT should provide new and important insights into the role of telomerase in cancer progression, and so help in the development of specific strategies for the therapeutic manipulation of TERT in human cancer. It was therefore decided to investigate whether telomerase regulates tumour invasion through regulating miRNA expression. To study exclusively the noncanonical functions of telomerase in this process, we generated a cell line model by stable transfection of the telomerase‐negative cell line, SAOS 2, with exogenous TERT. Taking advantage of the xenograft assay in zebrafish, we demonstrated that *TERT* overexpression increases the *in vivo* invasive capacity of hTERT‐SAOS2 compared with the parental cell line transfected with the empty plasmid (pBABE‐SAOS 2). However, their proliferation rate *in vitro* was unaltered.

As a starting point to study the triad TERT‐miRNA invasiveness, an miRNA array method was used to analyse changes in the miRNA expression in conditions of TERT overexpression. Surprisingly, only one statistically significant up‐regulated miRNA was identified – the oncomiR *miR500A*. The overexpression of *miR500A* in both pBABE‐SAOS 2 and hTERT‐SAOS 2 increased tumour invasion in xenografted zebrafish larvae, indicating its involvement in tumour invasion *per se*. Conversely, the inhibition of *miR500A* decreased tumour invasiveness but, interestingly, only in the tumour cells that express *TERT*, suggesting the existence of other players in the *in vivo* invasive capacity of SAOS 2 cells and demonstrating that TERT increases tumour invasiveness through *miR500A*. The inhibition of *miR500A* in another telomerase‐positive cell line, HeLa 1211, resulted in a similar outcome, confirming that the same mechanism operates in different tumour histotypes. The oncogenic activity of *miR500A* is not surprising, since high serum levels of *miR500A* are a diagnostic biomarker of hepatocellular carcinoma [21], and are associated with poor prognosis and poor overall survival rate in prostate cancer [22]. *miR500A* also closely correlated with malignant progression and poor survival in gastric cancer [23].

The regulation of miRNAs is poorly understood, due in part to the difficulty in predicting promoters from short conserved sequences. We localized *miR500A* in a cluster of eight miRNAs (*miR532, miR118, miR500A, miR362, miR501, miR500B, miR660* and *miR502*) in the intron 3 of the *CLCN5* gene, which is located in the short arm of the X chromosome (Xp11.23). Several studies have indicated that intronic miRNAs are not necessarily cotranscribed with their host gene, which suggests that they might have their own independent intronic promoters [24]. Our results showed that the expression of *CLCN5* is not affected by the presence of TERT, which suggests that the regulation of *miR500A* by TERT is not mediated through the regulation of its host gene promoter. As TERT has been reported to directly interact with TBE‐containing promoters [3, 4], we analysed the *miR500A* upstream sequence, and identified various TBE sequences upstream of *miR500A* and *miR532*. It was also found that TERT was able to increase the expression of a luciferase reporter driven by a 2‐Kb fragment upstream of *miR500A*, while, conversely, the inhibition of telomerase by siRNA decreased luciferase activity. These results were further confirmed by ChIP assays, where TERT was found to bind directly to the upstream region of *miR500A*, but not to the upstream region of *miR532*, even though this region also contained a TBE sequence. In addition, TERT regulated *miR362, miR500B* and *miR502*, all downstream of *miR500A*. However, the expression level of *miR532*, which is located upstream of *miR500A*, was unaffected by TERT. Taken together, these results demonstrate that TERT behaves as a TF that up‐regulates the expression of *miR500A* and all its downstream miRNAs.

According to the evolutionary model proposed by Chen and Rajewsky [25], a possible hypothesis to explain the TERT‐dependent regulation of the *miR*500 cluster, excluding the two miRNAs located upstream of *miR500A*, is that, although TERT originally could have interacted with a TSS (transcription start site) region in a potential intronic promoter to regulate the expression of the whole cluster, *miR500A* became the main effector of the cluster and developed its own TERT‐regulated promoter. At the same time, *miR532*, which is located upstream of this promoter, became a negative regulator of *TERT* as a compensatory mechanism in the fine‐tuning of TERT levels. However, this negative feedback needs to be confirmed with further experiments.

It has also been observed that genes involved in development have more TF‐binding sites and miRNA‐binding sites on average, meaning that genes of a higher *cis*‐regulation complexity are coordinately regulated by TFs at the transcriptional level and by miRNAs at the post‐transcriptional level [26]. Based on this observation, it is tempting to speculate that *TERT*, a crucial gene for life, regulates and is regulated by miRNAs of the same cluster. In support of this hypothesis, *miR500A* and all miRNAs downstream of the cluster are known to act as oncomiRs and are related to different cancer types, for example, *miR500A* in hepatocellular carcinoma, and gastric and breast cancer [27, 28], *miR362* in chronic myeloid leukaemia [29], *miR501* in gastric cancer [30], *miR660* in breast cancer [31] and *miR502* in colorectal and prostate cancer [32, 33]. Conversely, the miRNAs located upstream of *miR500A* act as tumour suppressors since *miR532* inhibits the expression of *TERT* in ovarian cancer, resulting in decreased cell proliferation and lower invasion capacity [34], and *miR188* is down‐regulated in oral squamous cell carcinoma [35]. In addition to being transcribed together, the different miRNAs of a cluster usually have the same function. To explore this possibility, we studied the effect of different components of the *miR500* cluster on the *in vivo* invasion capacity of SAOS 2 cells and, interestingly, only *miR500A* was seen to increase tumour invasion, while *miR532* had the opposite effect. Together, these data highlight the role played by TERT in the regulation of the *miR*500 cluster and the relevance of such crosstalk in cancer progression.

To catalogue the ability of TERT to regulate the *miR*500 cluster as a noncanonical function and its relevance in cancer invasion, we studied whether the absence of telomerase activity affects this activity and the invasiveness of tumour cells *in vivo* using two different approaches: a genetic approach using a DN‐TERT, and a pharmacological approach inhibiting either the TERT or *TERC* subunits. Surprisingly, neither the DN‐TERT nor the chemical inhibition of *TERC* with TAG 6 affected any function apart from telomerase activity *per se*, while BIBR 1532 reduced the expression of *miR500A* and, as a consequence, decreased tumour invasiveness *in vivo*. These results support the hypothesis of an extracurricular role for TERT in the transcriptional regulation of the *miR*500 cluster through its direct binding to the genomic DNA, helping to limit cancer progression and metastasis. Furthermore, they also point to the importance of choosing the right strategy when using telomerase inhibitors against cancer, since it can be more important to inhibit the extracurricular role of TERT by physically preventing its binding to DNA than inhibiting its enzymatic activity.

The *TargetScan* database and the *MetaCore* software functional annotations of predicted targets revealed crucial signalling pathways downstream of the TERT/*miR*500 cluster axis, including the Wnt/β‐catenin, NF‐κB and Hedgehog pathways, among others. We focused our attention on the Hh signalling pathway, since it is well established that its aberrant activation leads to enhanced proliferation and invasion of tumour cells. It was found that TERT‐induced *miR500A* mediated the down‐regulation of *PTCH1*, *GLI3* and *CUL3*, while *miR500A* directly targets the 3′UTR of *PTCH1*, promoting tumour invasiveness. Consistently, *GLI1* and *GLI2* mRNA levels increased, as they are negatively regulated by PTCH1 [36, 37]. In addition, PTCH1 inhibition increased the *in vivo* invasiveness of SAOS2 cells. The function of the receptor of Hh signalling pathway PTCH1 as a tumour suppressor is not surprising and has already been shown in other studies [36, 37]. The relevance of TERT *miR*500/Hh axis was confirmed in HeLa cells, where knocking down TERT resulted in decreased *miR500* levels, increased *PTCH1* levels and reduced *in vivo* invasiveness.

Using the data generated by TCGA Research Network, we found a strong positive correlation between *TERT* and *miR500A*, while *miR500A* expression was found to be negatively correlated with that of *PTCH1* in STAC and BLCA. High‐stage gastric cancer and negative PTCH1 staining have been identified as unfavourable risk factors for overall survival [38], and an important role for Hh signalling in bladder cancer growth and tumourigenicity has been described [39]. In addition, Shh signalling crosstalk with other signalling pathways during cancer development and progression, such as the Notch, Wnt and TGF‐β signalling pathways [40], which are also regulated by *miR500A* [27] and TERT [6], has also been described. Our results therefore support the idea that *miR500A* is also a good therapeutic target for fighting cancer.

## 5 Conclusion

**Fig. 7:**
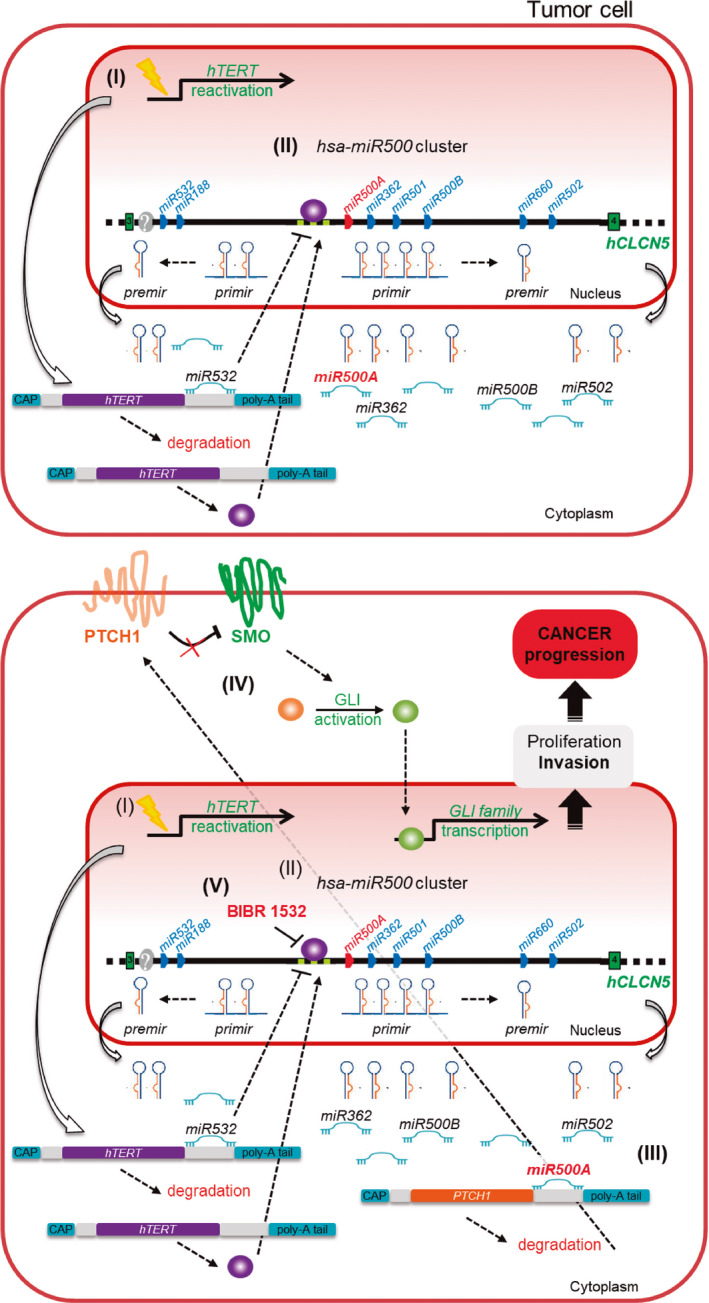
Extracurricular mechanism of TERT during invasion and tumour progression as seen by regulating *miR500A*. (I) Telomerase expression is reactivated in most tumours, and (II) TERT binds directly to the TBE sequences located at the promoter region of *miR500A*, resulting in the up‐regulation of *miR500A* and also the *miR*NAs located downstream of *miR500A*. As a compensatory mechanism, this regulation is fine‐tuned by the *miR532*, which acts as a negative regulator by repressing the *hTERT* mRNA. (III) The oncomiR *miR500A* represses post‐transcriptionally the mRNA of the tumour suppressor *PTCH1*, triggering a ligand‐independent aberrant Hedgehog signalling activation (IV) that contributes significantly to increase the invasiveness of tumour cells. (V) The chemical inhibition of TERT with BIBR 1532 could be a new strategy to fight cancer.

We have demonstrated for the first time that TERT is able to regulate the expression of specific microRNAs through its direct binding to TBE sequences in their promoter region. In particular, the TERT‐mediated up‐regulation of *miR500A* results in the post‐transcriptional repression of *PTCH1*, triggering a ligand‐independent aberrant Hh signalling that significantly increases tumour cell invasiveness in a zebrafish xenograft model (Fig. 7). This is a novel noncanonical telomerase function, since it is independent of telomerase activity, thus paving the way towards the development of new therapeutic strategies to fight cancer through the inhibition of noncanonical activities of TERT.

## Conflict of interest

The authors declare no conflict of interest.

## Author contributions

MLC conceived the study. FA‐P, VM and MLC designed the research. MBG, EM‐B, DG‐M, JG‐C, EB‐A and FA‐P performed the research. MB‐G, EM‐B, DG‐M, JG‐C, EB‐A, FA‐P, VM and MLC analysed the data. FA‐P and MLC wrote the manuscript with minor contribution from other authors.

## Ethics approval and consent to participate

The performed zebrafish experiments comply with the Guidelines of the European Union Council (86/609/EU). Experiments and procedures were performed as approved by the Bioethical Committee of the University Hospital ‘Virgen de la Arrixaca’ (HCUVA, Spain).

## Supporting information


**Fig. S1.** The expression of *TERT* increases the invasion capacity of the SAOS 2 cell line. (A) Quantification of the mRNA levels of *TERT* by real‐time RT‐qPCR after stable transfection of the SAOS 2 cell line with exogenous TERT, and (B) analysis of their *in vivo* invasive capacity using the zebrafish xenograft model and (C) Schematic of xenograft assay and analysis of cell invasion. (D) Images of representative zebrafish embryos in which cells have invaded or not their tissues (brain, muscle, tail…). Scale bar = 0.5 mm. Magnification of cells in the caudal region. In (A), each bar represents the mean ± SEM from triplicate samples. In (B), histogram represents the accumulated value of invasion percentage of the number of larvae stated in the figure for each treatment. ND, not detected; ns, not significant; ***P < 0.001 according to Mann Whitney test (A) and Fisher's exact test (B).

**Fig. S2.** The regulation of *miR500A* by TERT and its effect on the *in vivo* invasion capacity and the regulation of the Hedgehog signalling pathway also occur in other telomerase‐positive cells. Quantification of *TERT* (A) *miR500A* (B) and Hedgehog signalling pathway‐related genes (D–H) mRNA levels by real‐time RT‐qPCR, and determination of the *in vivo* invasion capacity (C) after specific inhibition of TERT with siRNA in the telomerase‐positive cell line HeLa 1211. In (A, B, D–H), each bar represents the mean ± SEM from triplicate samples. In (C), histograms represent the accumulative value of the invasion percentage of the number of larvae stated in the figure for each treatment. Graphs are representative (A, B, D–H) of three different experiments (*N* = 3). ns, not significant; *P < 0.05; **P < 0.01; ***P < 0.001 according to Student's *t*‐test (A, B, D–H) and Fisher's exact test (C).

**Fig. S3.** Reduction of *TERT* mRNA level using siRNA. Quantification of TERT mRNA level by real‐time RT‐qPCR after transfection of HEK293 cells with *TERT* or *siRNA‐TERT*. Each bar represents the mean ± SEM from triplicate samples. The graph is representative of two different experiments (*N* = 2). ***P < 0.001 according to ANOVA followed by Dunnett's multiple comparison test.

**Fig. S4.** Effect of the *miR*500 cluster members on *miR500A* and *TERT* expression levels. Overexpression of different miRNAs of the *miR*500 cluster by transient transfection in both pBABE‐SAOS 2 (A) and hTERT‐SAOS 2 (B, C). Quantification of *miR500A* (A, B) and *TERT* (C) mRNA levels by real‐time RT‐qPCR. Each bar represents the mean ± SEM from triplicate samples. Graphs are representative of three different experiments (*N* = 3). ns, not significant; **P < 0.01; ***P < 0.001 according to ANOVA followed by Dunnett's multiple comparison test.

**Fig. S5.** Specific chemical inhibition of telomerase activity. (A) Quantification of telomerase activity by Q‐TRAP after its specific chemical inhibition in hTERT‐SAOS 2 cells. (B) Quantification of the *miR500A* mRNA level by real‐time RT‐qPCR and (C) the percentage of invasion with a zebrafish xenograft assay in pBABE‐SAOS 2 cells. Each bar represents the mean ± SEM from triplicate samples (A, B). In (C), histogram represent the percentage of invasion of a number of larvae stated in the figure for each treatment. Graphs are the mean value (A) or representative (B) of three different experiments (*N* = 3) (A, B). ns, not significant; ***P < 0.001 according to ANOVA followed by Tukey's multiple comparison test (A, B) and Fisher's exact test (C).

**Fig. S6.** (A) The top 10 biological processes associated with endogenous *miR*500A‐binding sites according to MetaCore. (B) Alignment between the seed sequence of the *miR500A* (underlined, in red) and the 3′UTR of *PTCH1*, *GLI3* and *CUL3*, from the Hedgehog signaling pathway.

**Fig. S7.** Quantification of *miR500A*, *GLI1* and *GLI2* levels. Quantification of the mRNA level of *miR500A* (A), *GLI1* (B) and *GLI2* (C) by real‐time RT‐qPCR in TERT overexpression conditions and after cotransfection of the pBABE‐SAOS 2 cells with the *premiR500A* and a reporter plasmid containing the wild‐type (wt) or a mutated (mut) 3′UTR of *PTCH1* (D). Each bar represents the mean ± SEM from triplicate samples and graphs are representative of three different experiments (*N* = 3). **P < 0.01; ***P < 0.001; ****P < 0.0001 according to Student's *t*‐test (A–C) and ANOVA followed by Bonferroni's multiple comparison test (D).

**Table S1.** Gene accession numbers and primer sequences used for gene expression analysis.
**Table S2.** Sequence of the different primers used for the analytical qPCR of ChIP.

## Data Availability

The data that support the findings are available from the corresponding author (marial.cayuela@carm.es) upon reasonable request.
