# Supplementary figures and images for: Telomerase reverse transcriptase activates transcription of miR500A to inhibit Hedgehog signalling and promote cell invasiveness
Source: Mol Oncol. 2021 May 2;15(7):1818–34. doi: 10.1002/1878-0261.12943 (PMC8253104; doi:10.1002/1878-0261.12943)

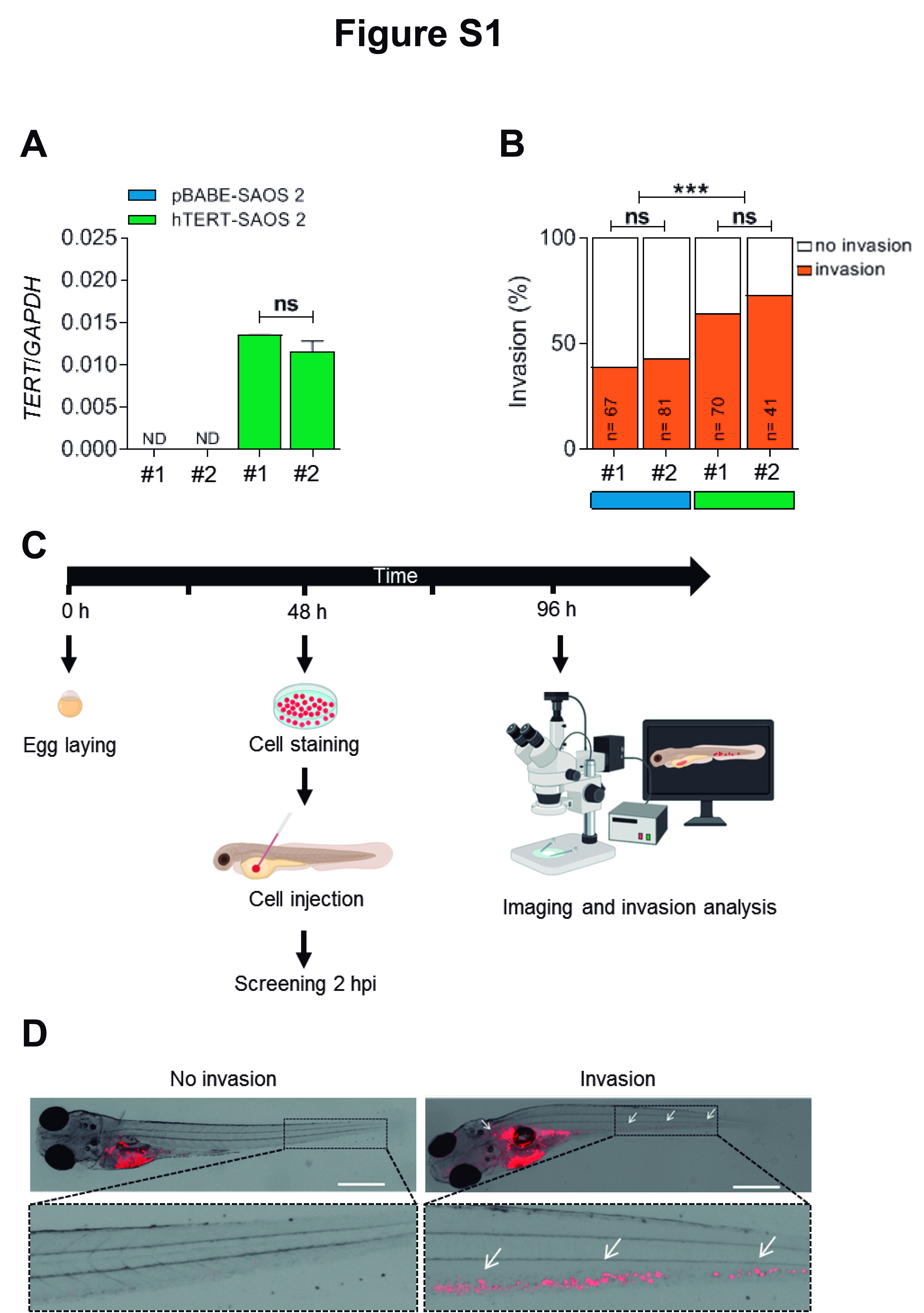

Supplement: Supplementary file 1 — Fig. S1. The expression of TERT increases the invasion capacity of the SAOS 2 cell line. (A) Quantification of the mRNA levels of TERT by real‐time RT‐qPCR after stable transfection of the SAOS 2 cell line with exogenous TERT, and (B) analysis of their in vivo invasive capacity using the zebrafish xenograft model and (C) Schematic of xenograft assay and analysis of cell invasion. (D) Images of representative zebrafish embryos in which cells have invaded or not their tissues (brain, muscle, tail…). Scale bar = 0.5 mm. Magnification of cells in the caudal region. In (A), each bar represents the mean ± SEM from triplicate samples. In (B), histogram represents the accumulated value of invasion percentage of the number of larvae stated in the figure for each treatment. ND, not detected; ns, not significant; ***P < 0.001 according to Mann Whitney test (A) and Fisher's exact test (B). [file MOL2-15-1818-s002.jpg]

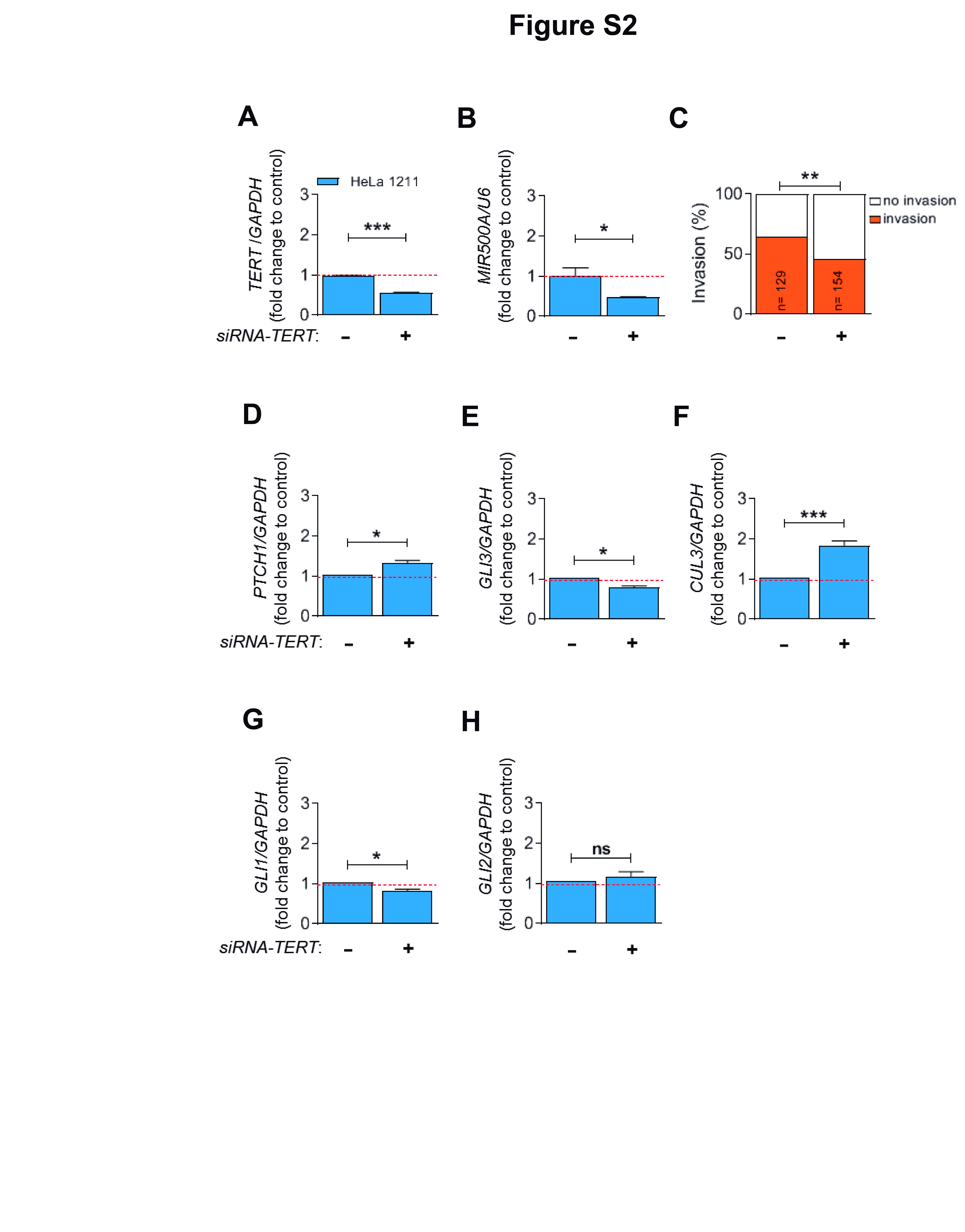

Supplement: Supplementary file 2 — Fig. S2. The regulation of miR500A by TERT and its effect on the in vivo invasion capacity and the regulation of the Hedgehog signalling pathway also occur in other telomerase‐positive cells. Quantification of TERT (A) miR500A (B) and Hedgehog signalling pathway‐related genes (D–H) mRNA levels by real‐time RT‐qPCR, and determination of the in vivo invasion capacity (C) after specific inhibition of TERT with siRNA in the telomerase‐positive cell line HeLa 1211. In (A, B, D–H), each bar represents the mean ± SEM from triplicate samples. In (C), histograms represent the accumulative value of the invasion percentage of the number of larvae stated in the figure for each treatment. Graphs are representative (A, B, D–H) of three different experiments (N = 3). ns, not significant; *P < 0.05; **P < 0.01; ***P < 0.001 according to Student's t‐test (A, B, D–H) and Fisher's exact test (C). [file MOL2-15-1818-s007.jpg]

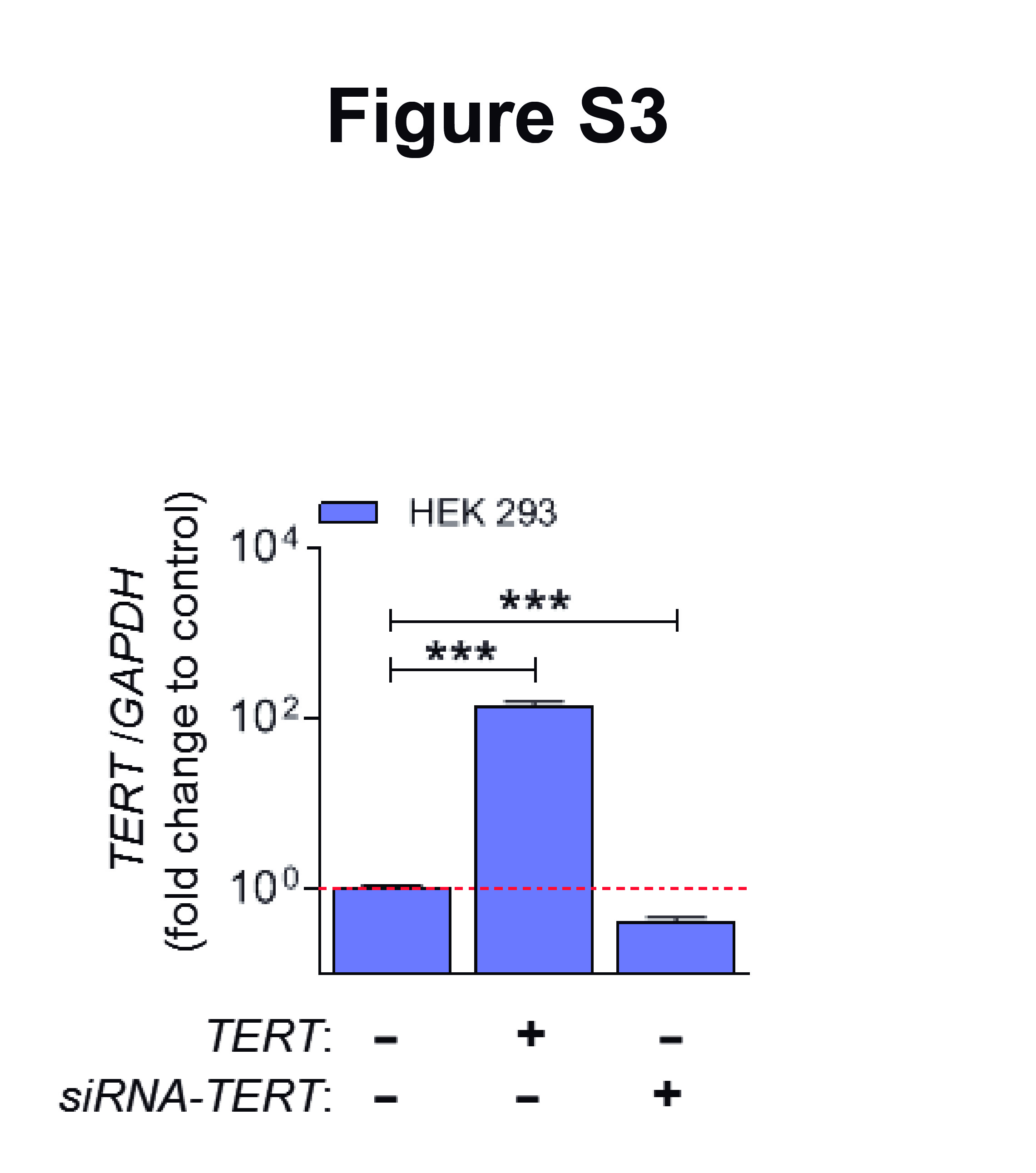

Supplement: Supplementary file 3 — Fig. S3. Reduction of TERT mRNA level using siRNA. Quantification of TERT mRNA level by real‐time RT‐qPCR after transfection of HEK293 cells with TERT or siRNA‐TERT. Each bar represents the mean ± SEM from triplicate samples. The graph is representative of two different experiments (N = 2). ***P < 0.001 according to ANOVA followed by Dunnett's multiple comparison test. [file MOL2-15-1818-s003.jpg]

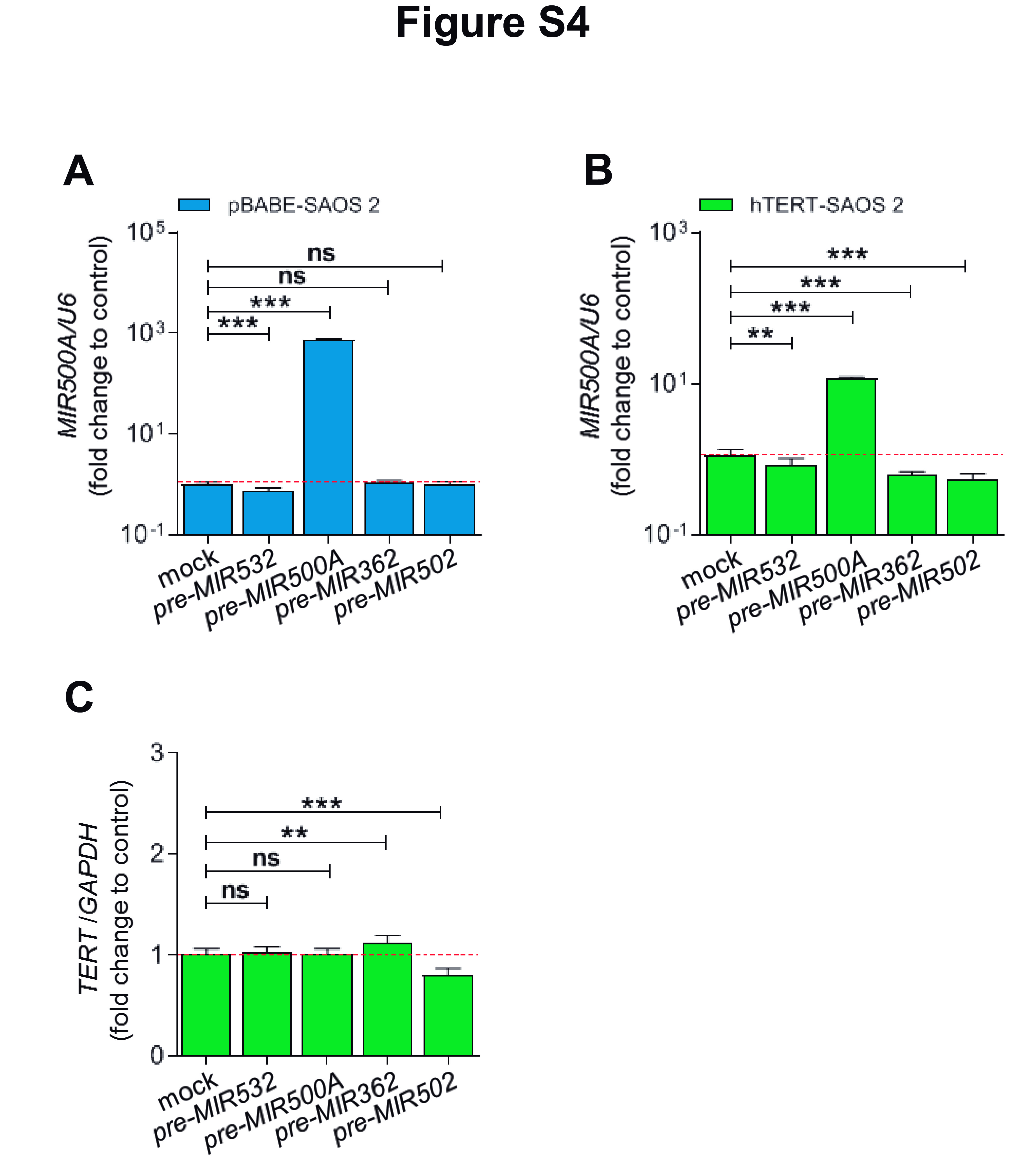

Supplement: Supplementary file 4 — Fig. S4. Effect of the miR500 cluster members on miR500A and TERT expression levels. Overexpression of different miRNAs of the miR500 cluster by transient transfection in both pBABE‐SAOS 2 (A) and hTERT‐SAOS 2 (B, C). Quantification of miR500A (A, B) and TERT (C) mRNA levels by real‐time RT‐qPCR. Each bar represents the mean ± SEM from triplicate samples. Graphs are representative of three different experiments (N = 3). ns, not significant; **P < 0.01; ***P < 0.001 according to ANOVA followed by Dunnett's multiple comparison test. [file MOL2-15-1818-s008.jpg]

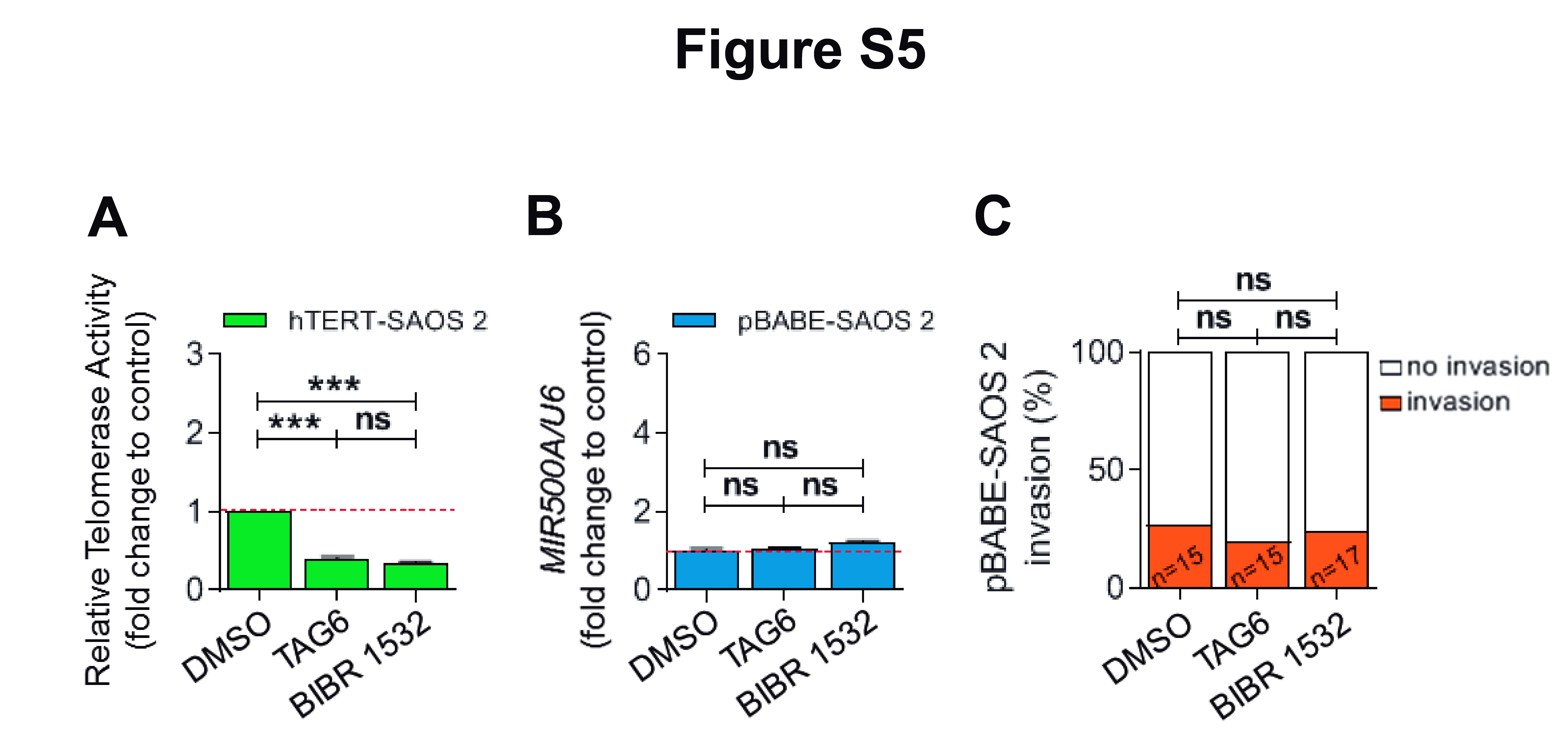

Supplement: Supplementary file 5 — Fig. S5. Specific chemical inhibition of telomerase activity. (A) Quantification of telomerase activity by Q‐TRAP after its specific chemical inhibition in hTERT‐SAOS 2 cells. (B) Quantification of the miR500A mRNA level by real‐time RT‐qPCR and (C) the percentage of invasion with a zebrafish xenograft assay in pBABE‐SAOS 2 cells. Each bar represents the mean ± SEM from triplicate samples (A, B). In (C), histogram represent the percentage of invasion of a number of larvae stated in the figure for each treatment. Graphs are the mean value (A) or representative (B) of three different experiments (N = 3) (A, B). ns, not significant; ***P < 0.001 according to ANOVA followed by Tukey's multiple comparison test (A, B) and Fisher's exact test (C). [file MOL2-15-1818-s005.jpg]

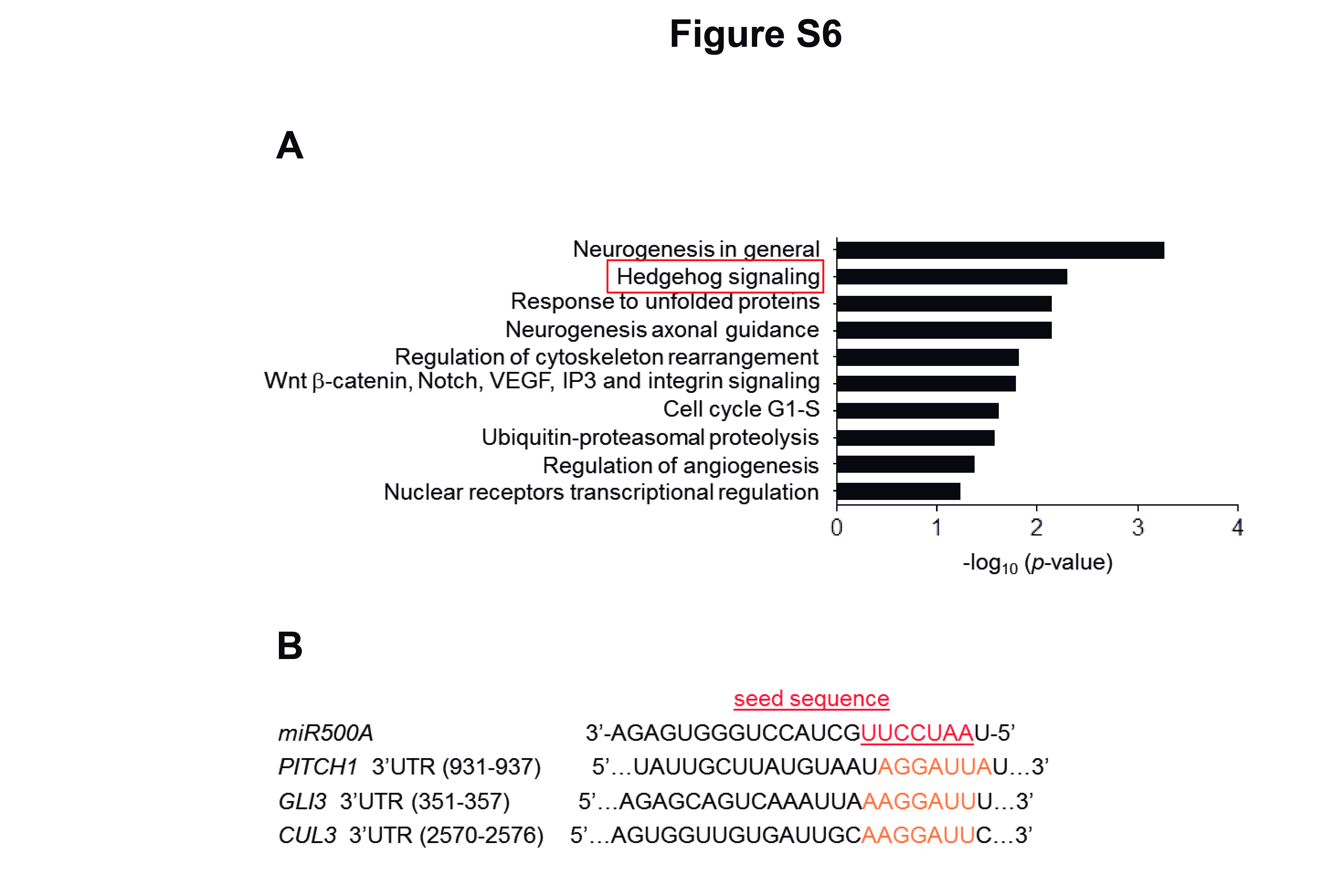

Supplement: Supplementary file 6 — Fig. S6. (A) The top 10 biological processes associated with endogenous miR500A‐binding sites according to MetaCore. (B) Alignment between the seed sequence of the miR500A (underlined, in red) and the 3′UTR of PTCH1, GLI3 and CUL3, from the Hedgehog signaling pathway. [file MOL2-15-1818-s004.jpg]

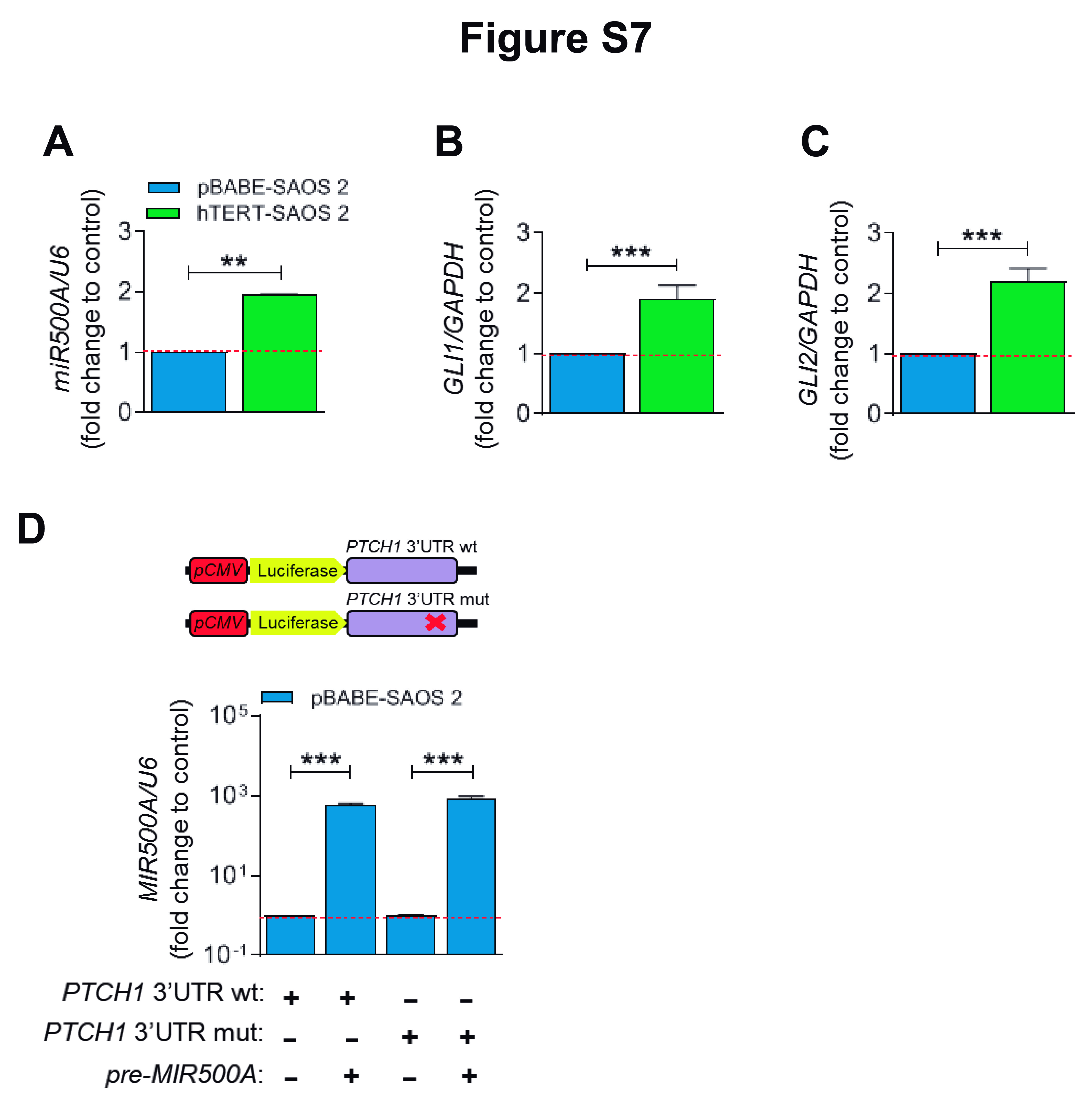

Supplement: Supplementary file 7 — Fig. S7. Quantification of miR500A, GLI1 and GLI2 levels. Quantification of the mRNA level of miR500A (A), GLI1 (B) and GLI2 (C) by real‐time RT‐qPCR in TERT overexpression conditions and after cotransfection of the pBABE‐SAOS 2 cells with the premiR500A and a reporter plasmid containing the wild‐type (wt) or a mutated (mut) 3′UTR of PTCH1 (D). Each bar represents the mean ± SEM from triplicate samples and graphs are representative of three different experiments (N = 3). **P < 0.01; ***P < 0.001; ****P < 0.0001 according to Student's t‐test (A–C) and ANOVA followed by Bonferroni's multiple comparison test (D). [file MOL2-15-1818-s001.jpg]
