# Supplementary material for: Telomerase reverse transcriptase activates transcription of miR500A to inhibit Hedgehog signalling and promote cell invasiveness
Source: Mol Oncol. 2021 May 2;15(7):1818–34. doi: 10.1002/1878-0261.12943 (PMC8253104; doi:10.1002/1878-0261.12943)
Supplement: Supplementary file 8 — Table S1. Gene accession numbers and primer sequences used for gene expression analysis. Table S2. Sequence of the different primers used for the analytical qPCR of ChIP. [file MOL2-15-1818-s006.docx]

**Table I: Gene accession numbers and primer sequences used for gene expression analysis.**

| **Gene** | **Gene accession number** | **Primer name** | **Sequence (5’-3’)** |
| --- | --- | --- | --- |
| *GAPDH* | NM_002046.6 | QT01192646 | QuantiTect Primer Assay (QIAGEN) |
| *U6* | NR_004394.1 | F  R | GAGGGCCTATTTCCCATGATT  TAATTAGAATTAATTTGACT |
| *TERT* | NM_198253.2 | F  R | TGACACCTCACCTCACCCAC  CACTGTCTTCCGCAAGTTCAC |
| *miR-532* | MIMAT0002888 | F | CAUGCCUUGAGUGUAGGACCGU |
| *miR-500a* | MIMAT0004773 | F | UAAUCCUUGCUACCUGGGUGAGA |
| *miR-362* | MIMAT0000705 | F | AAUCCUUGGAACCUAGGUGUGAGU |
| *miR-500b* | MIMAT0016925 | F | AAUCCUUGCUACCUGGGU |
| *miR-502* | MIMAT0002873 | F | AUCCUUGCUAUCUGGGUGCUA |
| *PTCH1* | NM_001083602.2 | QT00075824 | QuantiTect Primer Assay (QIAGEN) |
| *GLI3* | NM_000168.5 | QT00045682 | QuantiTect Primer Assay (QIAGEN) |
| *CUL3* | NM_003590.4 | QT00051296 | QuantiTect Primer Assay (QIAGEN) |
| *GLI1* | NM_005269.3 | F  R | AGATGAATCACCAAAAAGGG  ATATCACCTTCCAAGGGTTC |
| *GLI2* | NM_001371271.1 | F  R | TACCAGCAGATTCTGAGC  CTCTGCTTGTTCTGGTTG |

**Table II: Sequence of the different primers used for the analytical qPCR of ChIP.**

| **Promoter region** | **Primer name** | **Sequence (5’-3’)** |
| --- | --- | --- |
| **Controls** |  |  |
| ***Intron_GAPDH*** | **F**  **R** | **AATGGGCAGCCGTTAGGAAA**  **AAAAGCATCACCCGGAGGAG** |
| ***TBE_cMyc*** | **F**  **R** | **CGTTTTCCTCCTTATGCCTCTATC**  **GTACCAGGCTGCAGGGCGCCTCGCT** |
| **Transcription points in the cluster** |  |  |
| ***Up_miR-532 (upstream)*** | **F**  **R** | **TGCACACATGCTGGGGATAC**  **GAGACAGCTCACTGCCCTTT** |
| ***Up_miR-500a (upstream)*** | **F**  **R** | **CACACAAAGCATTTGCGGGA**  **CACACAAAGCATTTGCGGGA** |
